# Distinct urinary glycoprotein signatures in prostate cancer patients

**DOI:** 10.18632/oncotarget.26005

**Published:** 2018-09-04

**Authors:** Rebeca Kawahara, Fabio Ortega, Livia Rosa-Fernandes, Vanessa Guimarães, Daniel Quina, Willian Nahas, Veit Schwämmle, Miguel Srougi, Katia R.M. Leite, Morten Thaysen-Andersen, Martin R. Larsen, Giuseppe Palmisano

**Affiliations:** ^1^ Instituto de Ciências Biomédicas, Departamento de Parasitologia, Universidade de São Paulo, USP, São Paulo, Brazil; ^2^ Laboratório de Investigação Médica da Disciplina de Urologia da Faculdade de Medicina da USP, LIM55, São Paulo, Brazil; ^3^ Department of Biochemistry and Molecular Biology, University of Southern Denmark, Odense, Denmark; ^4^ Instituto do Câncer do Estado de São Paulo, ICESP, São Paulo, Brazil; ^5^ Department of Molecular Sciences, Macquarie University, Sydney, NSW, Australia

**Keywords:** prostate cancer, urine, glycopeptide, TMT-labeling, glycoproteomics

## Abstract

Novel biomarkers are needed to complement prostate specific antigen (PSA) in prostate cancer (PCa) diagnostic screening programs. Glycoproteins represent a hitherto largely untapped resource with a great potential as specific and sensitive tumor biomarkers due to their abundance in bodily fluids and their dynamic and cancer-associated glycosylation. However, quantitative glycoproteomics strategies to detect potential glycoprotein cancer markers from complex biospecimen are only just emerging. Here, we describe a glycoproteomics strategy for deep quantitative mapping of *N*- and *O*-glycoproteins in urine with a view to investigate the diagnostic value of the glycoproteome to discriminate PCa from benign prostatic hyperplasia (BPH), two conditions that remain difficult to clinically stratify. Total protein extracts were obtained, concentrated and digested from urine of six PCa patients (Gleason score 7) and six BPH patients. The resulting peptide mixtures were TMT-labeled and mixed prior to a multi-faceted sample processing including hydrophilic interaction liquid chromatography (HILIC) and titanium dioxide SPE based enrichment, endo-/exoglycosidase treatment and HILIC-HPLC pre-fractionation. The isolated *N*- and *O*-glycopeptides were detected and quantified using high resolution mass spectrometry. We accurately quantified 729 *N*-glycoproteins spanning 1,310 unique N-glycosylation sites and observed 954 and 965 unique intact *N*- and *O*-glycopeptides, respectively, across the two disease conditions. Importantly, a panel of 56 intact N-glycopeptides perfectly discriminated PCa and BPH (ROC: AUC = 1). This study has generated a panel of intact glycopeptides that has a potential for PCa detection.

## INTRODUCTION

Prostate cancer (PCa) is the most common non-cutaneous cancer and the second leading cause of cancer-related death in men [1]. Although blood levels of prostate specific antigen (PSA) is the most common diagnostic marker used for PCa screening, its low specificity has raised concerns regarding patient over-diagnosis and overtreatment [2]. For example, elevated serum PSA levels are associated with benign prostatic hyperplasia (BPH), a common age-related benign male condition, that shares similar clinical symptoms with malignant prostate disorders [3]. The urgent need for novel biomarkers that can effectively stratify BPH and PCa patients has prompted scientists to search for PCa-specific biomolecules in bodily fluids.

Urine is an attractive excreted body fluid for PCa biomarker discovery, since it is readily obtained in a non-invasive manner and may, due to the proximity to the prostate, carry biomolecular signatures that reflect the aberrant biochemical processes occurring within the prostate during PCa development and progression [4, 5]. Mass spectrometry-based proteomics has been instrumental in the discovery of several candidate biomarkers in urine from PCa patients [6]. For example, Ahmad *et al.* [7] reported unusually low urinary levels of fibronectin and TP53INP2 in PCa patients compared to BPH patients. Jedinak *et al.* [8] reported a panel of differentially expressed proteins in PCa compared to BPH urine including β-2-microglobulin (β2M), pepsinogen 3 (PGA3), and mucin 3 (MUC3). These examples illustrate that sensitive quantitation of aberrant protein signatures directly in urine is now technically feasible using modern LC-MS/MS technologies. However, disappointingly few of the proposed biomarker candidates for PCa diagnosis have been validated across multiple large PCa patient cohorts and none is, to the best of our knowledge, implemented in the clinic for PCa screening.

Protein glycosylation is increasingly being recognized as a modification with a great potential for cancer diagnostics [9–15]. Aberrations in the glycosylation machinery leading to “tumor-specific” glycan structures or epitopes are universal feature of malignant transformation and tumor progression [16–18]. Vermassen *et al.* showed that glycan profiling of urinary proteins could discriminate PCa from BPH patients [19]. In that specific study, *N*-glycans were released from urinary glycoproteins, labeled with with 8-aminopyrene-1,3,6-trisulphonic acid and analyzed by a multi-capillary electrophoresis-based sequencer. However, no site-specific information of the aberrant glycans that showed core fucosylation features associated with PCa could be derived from this glycomics focused approach. In another study, core fucosylation was observed as an enriched glyco-feature in PCa cell lines derived from metastatic cells relative to non-tumorigenic prostate cells [20]. It remains unclear if such PCa cell lines accurately reflect the glyco-phenotype of PCa tissue.

Liu *et al.* used mass spectrometry to explore former *N-*glycosylated peptides (hereafter de-*N*-glycosylated peptides or de-*N*-glycopeptides) in normal prostate, non-aggressive, aggressive and metastatic PCa tissues [21]. In total, 1,430 *N-*glycosylation sites were identified and some glycoproteins were proposed as biomarker candidates for PCa aggressiveness including N-acylethanolamine acid amidase and protein tyrosine kinase 7. Cima *et al.* applied label-free quantitative proteomics to measure perturbations in the prostate and serum glycoproteome of Pten conditional knockout mice relative to matching wildtype mice. In that study, promising candidate biomarkers, such as thrombospondin-1 (THBS1), tissue inhibitor of metalloproteinase 1 (TIMP-1), complement factor H (CFH), and prolow-density lipoprotein receptor-related protein 1 (LRP-1), were further validated by quantitative selected reaction monitoring (SRM) and ELISA in the sera of PCa and BPH patients [22]. Shah *et al.* showed altered fucosylation in the androgen-independent PCa cell line PC3 compared to the androgen-dependent PCa cell LNCap line using glycoproteomics. A total of 1,810 unique *N-*glycosylation sites from 653 *N-*glycoproteins were identified; 176 glycoproteins were present in different levels between the two cell lines [20]. Core fucosylation was observed as an enriched glyco-feature in PCa cell lines derived from metastatic cells relative to non-tumorigenic prostate cells [20]. However, it remains unclear if such PCa cell lines accurately reflect the glyco-phenotype of PCa tissue.

Collectively, these studies have demonstrated the immense potential of present day mass spectrometry to map the glycoproteome and identify glycoproteins with untapped diagnostic potential to report on malignant transformation. However, to the best of our knowledge, no studies have to date profiled site-specific protein glycosylation features directly in urine from PCa and relevant control donors to investigate the potential of using aberrant glycoproteome signatures as accurate molecular reporters of PCa development and progression.

Despite of the impressive progress achieved in the now streamlined genome and proteome sequencing methods over the past decades, glycoproteomics technologies remain under-developed and are still only utilized in specialist laboratories [23, 24]. Technical challenges are still preventing or at least discouraging more general Proteomics focused laboratories. Key steps in the glycoproteomics workflow including glycopeptide enrichment, liquid chromatography–mass spectrometry (LC-MS/MS) acquisition and computational data analysis are still common bottlenecks that limit the identification rate and the quantitative accuracy when investigating the extensive micro- and macro-heterogeneity inherently associated with the *N-* and *O*-glycoproteome [25, 26].

We recently described a method for site-specific characterization of the *N-*glycan composition, peptide carrier and glycosylation sites from urinary glycoproteins and endogenous glycopeptides of healthy donors [27]. Hydrophilic interaction liquid chromatography (HILIC) SPE-enriched glycopeptides were analyzed in their intact form by LC-MS/MS; in total, 472 unique *N-*glycosylation sites and 303 intact *N-*glycopeptides from 256 urinary *N*-glycoproteins were identified. Following on our previous work in urinary glycoproteomics, we here set out to expand our coverage of the urinary glycoproteome by combining complementary glycopeptide enrichment and pre-fractionation strategies with exo- and endo-glycosidase digestion prior to LC-MS/MS. Importantly, we use isobaric labels to facilitate accurate quantitative glycoproteomics between and within donor groups to more effectively investigate the potential of intact glycopeptides as signatures for PCa detection and precise stratification from BPH conditions.

The innovative aspects of this study are summarized as follows: 1) we present a mass spectrometry-based method for a highly sensitive site-specific and quantitative characterization of the *N-* and *O-*glycosylation decorating urinary proteins, 2) we used this method to obtain the largest map of human urinary glycoproteome to date including identifying glycosylated proteins, glycosylation sites and site-specific glycan compositions, and importantly 3) this method also showed that a panel of PCa-associated glycoproteins, as measured by intact glycopeptides, can be used to discriminate urine from PCa and BPH. Technical aspects pertaining to this glycoproteomics strategy and the panel of PCa-associated urinary glycoproteins are here discussed in the context of PCa detection and prostate biology.

## RESULTS

### Quantitative glycoproteomics enable exploration of the urinary *N*- and *O*-glycoproteome

**Figure 1:**
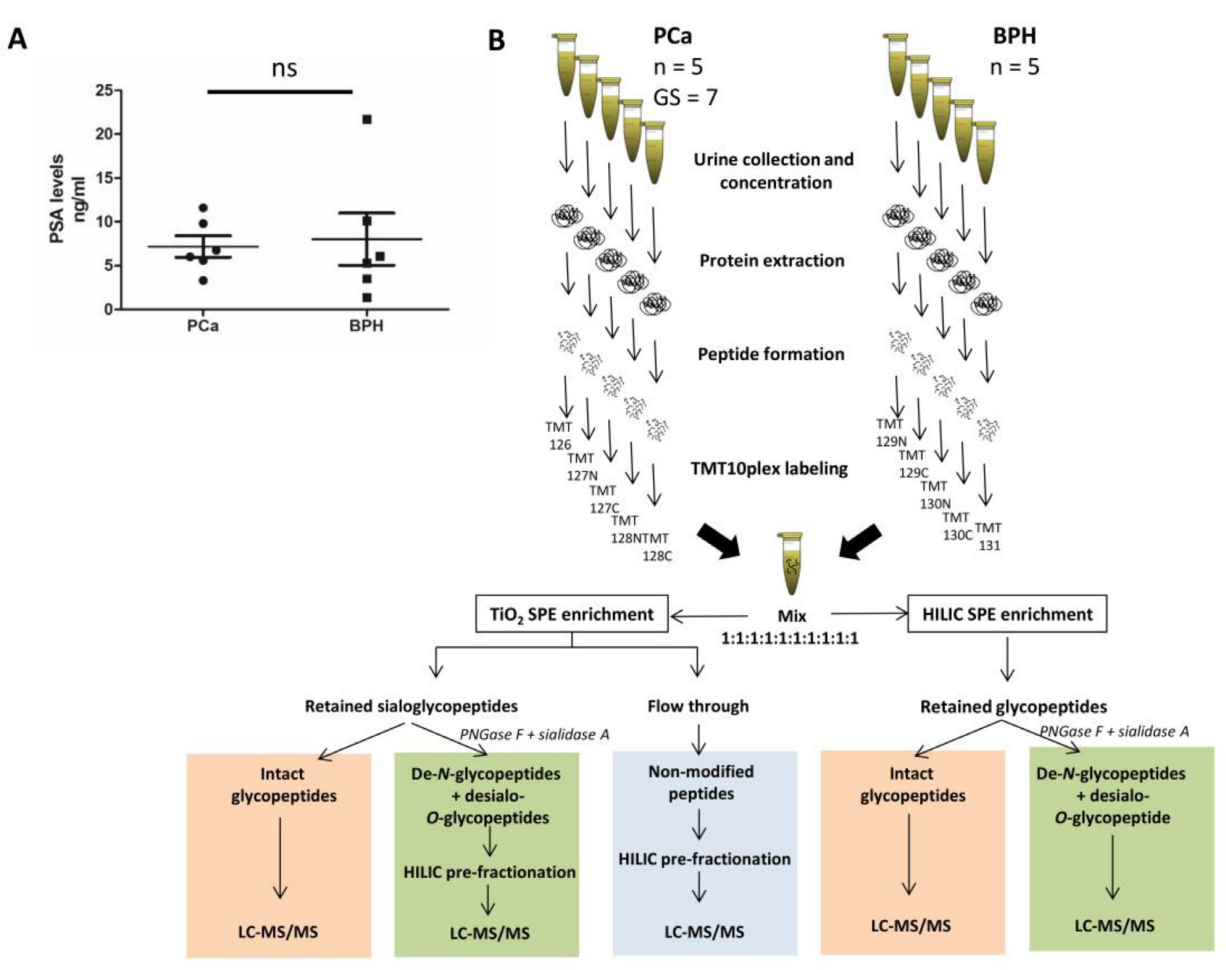
TMT label-assisted quantitative glycoproteomics of urinary glycoprotein from PCa and BPH patients (**A**) The serum PSA levels of PCa (*n =* 6) and BPH (*n =* 6) patients showed no statistical difference (*p >* 0.05, unpaired two tailed *t*-test). (**B**) Overview of the quantitative glycoproteomics workflow employed in this study. Peptides were generated from extracted urinary proteins from each patient, labelled with isobaric TMT tags and mixed 1:1 (w:w). The glycopeptides were selectively enriched using either TiO_2_ or HILIC SPE and the resulting fractions were either analyzed directly or after simultaneous endo- and exoglycosidase treatment by LC-MS/MS. Former *N*-glycosylated peptides generated by PNGase F (referred to as de-*N*-glycosylated peptides) and desialo-*O*-glycosylated peptides generated by sialidase and non-modified peptides were pre-fractionated using off-line HILIC HPLC; the resulting fractions were analyzed by LC-MS/MS. Different information was obtained from the analysis of intact glycopeptides (red), de-*N*-glycopeptides (green) and non-modified peptides (blue).

Urine samples (∼2 ml) from six PCa and six BPH patients were obtained and investigated in this study. The PCa group included only patients with clinically validated adenocarcinoma with Gleason score 7 (GS 7), which denotes intermediate grade PCa relative to GS 6 representing low-risk PCa suitable for an active surveillance program, and the more advanced GS 8–10 representing high-risk PCa cases that generally are referred to immediate treatment. The serum PSA levels of the PCa patient group were 7.17 ± 3.02 ng/ml (*n =* 6) and 8.02 ± 7.30 ng/ml in the BPH patient group (*n =* 6). As expected, no significant difference was observed in the serum PSA levels between these two patient groups (*p* ≥ 0.05, *n =* 6, unpaired two-tailed *t*-test, Figure 1A) highlighting the shortcoming of this biomarker to accurately stratify PCa and BHP patients. Lower yields of total urinary protein were observed from two biological replicates from both the PCa and BPH groups; hence, these protein-poor samples were combined (1:1, w/w) forming five biological replicates (*n =* 5) from each condition for comparative glycoproteomics.

Given the reasonably large protein starting material (∼100 µg/replicate), we applied a multi-faceted quantitative LC-MS/MS-based glycoproteomics strategy to obtain a deep coverage of the urinary glycoproteome and investigate for differentially abundant glycoproteins in PCa and BPH urine. In short, after protein extraction, concentration and tryptic digestion of the 10 samples, the resulting peptide mixtures were labeled with an isobaric amine-reactive TMT10plex – peptides from the five PCa samples were labeled with TMT 126, 127N, 127C, 128N and 128C and peptides from the five BPH samples were labeled with TMT 129N, 129C, 130N, 130C and 131. All TMT-labelled peptide samples were then mixed in 1:1 (w/w) relationships prior to a multi-faceted glycopeptide enrichment and pre-fractionation sample processing (Figure 1B).

The glycopeptide enrichment was performed using two complementary solid phase extraction (SPE) strategies i.e. titanium dioxide (TiO_2_) SPE and HILIC SPE enrichment, to reach the deepest possible coverage of the urinary glycoproteome. The sialic acid-retaining TiO_2_ SPE [28, 29] was included since we previously showed that urinary *N*-glycoproteins are highly sialylated [27]. HILIC SPE is widely recognized to capture all glycopeptides displaying a minimum degree of hydrophilicity regardless of the nature of their conjugated glycan structure [30, 31]; however, this enrichment method may come short of quantitatively capturing *O*- and *N*-glycopeptides of very low hydrophilicity (unpublished observation). The TiO_2_ and HILIC SPE enriched glycopeptides were 1) analyzed directly in their intact form by high resolution LC-MS/MS on a Orbitrap Q-Exactive HF with HCD fragmentation, which provided information of the peptide carrier and site-specific glycan heterogeneity, or 2) treated simultaneously with *N*-glycosidase F (PNGase F) and sialidase A to generate de-*N*-glycosylated and desialo-*O*-glycopeptides that are less challenging to characterize by LC-MS/MS. Combining the latter approach with a similar Orbitrap Q-Exactive HF HCD based acquisition, provided *N-*glycosylation site information and site-specific glycan information of the *O*-glycosylation. In addition, non-modified peptides that did not bind to the TiO_2_ SPE column (“flow through”) and PNGase F/sialidase-treated glycopeptides were pre-fractionated using off-line HILIC HPLC and the resulting fractions were analyzed by Orbitrap Q-Exactive HF HCD-MS/MS (Figure 1B).

### Complementary TiO_2_ and HILIC SPE-based glycopeptide enrichment provide deep coverage of the urinary glycoproteome

Even without peptide pre-fractionation, the combined use of TiO_2_ and HILIC SPE facilitated the identification of 630 unique *N-*linked glycosylation sites belonging to 361 *N-*glycoproteins across the urine samples (Supplementary Figure 1A, and Supplementary Tables 1–2). Partial overlap between the TiO_2_ and HILIC SPE enrichment approaches were observed when measured by the proportion of unique *N-*glycosylation sites (48%, 304/630) and the corresponding glycoproteins (58%, 211/361) detected in both sample preparation techniques (Supplementary Figure 1B–1C). However, when assessed by the identified intact glycopeptides, the enrichment strategies displayed high complementarity: From a combined total of 954 *N-* and 210 *O-*glycopeptides identified with high peptide confidence (0% peptide FDR Pep-2D) [32] (Supplementary Figure 1D and Supplementary Tables 3–6), the degree of unique *N-* and *O-*glycopeptides identified from both the TiO_2_ and HILIC SPE preparations was 23% (216/954) and 10% (20/210), respectively (Supplementary Figure 1E–1F).

Due to the unbiased enrichment of the hydrophilic glycopeptides, HILIC SPE showed, as expected, a higher capture efficiency of *N-*glycopeptides compared to TiO_2_ SPE. In contrast, TiO_2_ retained a greater number of *O-*glycopeptides compared to HILIC SPE indicating a high degree of sialylation of the *O-*glycopeptides and an inability of HILIC SPE to retain such lowly hydrophilic glycopeptides.

We also investigated the glycosylation features of the TiO_2_ and HILIC SPE enriched intact *N-*glycopeptides (Supplementary Figure 1G, Supplementary Tables 3–4) and *O-*glycopeptides (Supplementary Figure 1H, Supplementary Tables 5–6). In agreement with our previous study [27], the urinary *N*-glycoproteins were predominantly carrying complex and hybrid type *N*-glycans with a high level of fucosylation and/or sialylation accounting for ∼85% of all identified intact *N-*glycopeptides (Supplementary Figure 1G). Sialylated *O-*glycopeptides were also abundant (∼70%) and were, as expected, efficiently enriched using TiO_2_ SPE (Supplementary Figure 1H). Collectively, these data clearly demonstrated that the parallel usage of HILIC and TiO_2_ SPE is highly beneficial to increase the coverage of the urinary *N*- and *O*-glycoproteome.

### HILIC HPLC pre-fractionation enhances the coverage of the urinary glycoproteome

**Figure 2:**
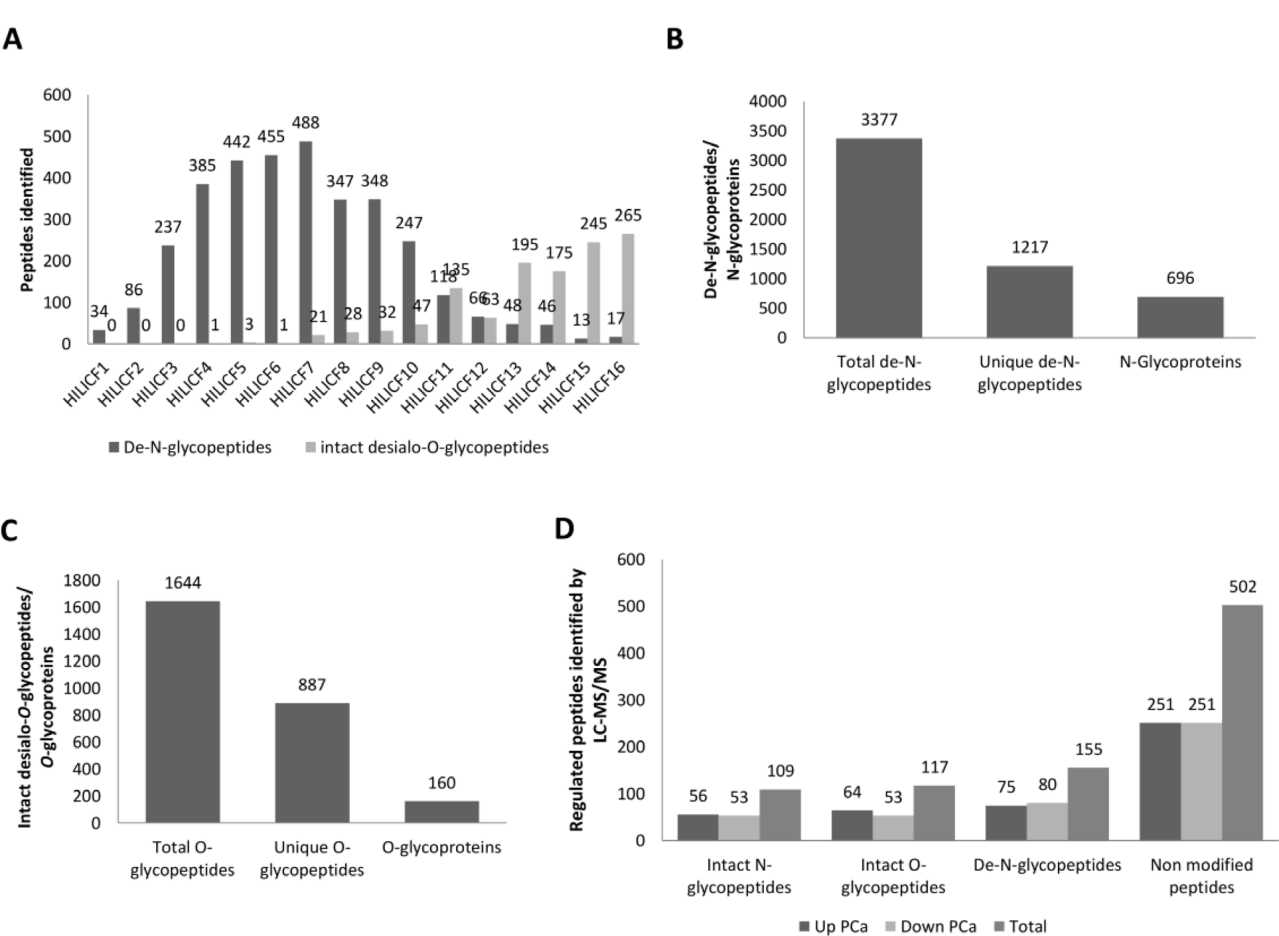
Deep coverage of the urinary *N*- and *O*-glycoproteome using HILIC HPLC pre-fractionation reveals quantitative glycoproteome differences in PCa and BPH (**A**) Distribution of de-*N*-glycopeptides and intact desialo-*O-*glycopeptides identified across the fractions arising from the HILIC HPLC pre-fractionation of TiO_2_ SPE enriched peptides of urinary proteins. (**B**) Total and unique de-*N*-glycopeptides/*N*-glycoproteins and (**C**) intact desialo-*O-*glycopeptides/*O*-glycoproteins after HILIC HPLC pre-fractionation. (**D**) Overview of the differentially abundant intact *N-*glycopeptides (from TiO_2_ and HILIC SPE enrichment), desialo-*O-*glycopeptides, de-*N*-glycosylated peptides and non-modified peptides identified in the glycoproteome of PCa relative to BHP urine.

In attempts to reach an even greater coverage of the urinary glycoproteome, off-line HILIC HPLC was implemented in the workflow to pre-fractionate the TiO_2_ SPE retained glycopeptide fraction. Endo- (PNGase F) and exo- (broad specificity sialidase) glycosidase treatments were also introduced to render the HILIC peptide fractions more amendable to LC-MS/MS detection; it is commonly known that such de-*N*-glycosylated and desialo-*O*-glycopeptides are easier to identify than their native counterparts, albeit with less structural information obtained [33, 34]. Interesting, the de-*N-*glycosylated peptides eluted early in the HILIC HPLC gradient and were efficiently separated from the highly retained desialo-*O-*glycopeptides (Figure 2A). When combined with Orbitrap Q-Exactive HF HCD-MS/MS detection, the HILIC HPLC pre-fractionation facilitated an unprecedented coverage of the urinary glycoproteome by the identification of a total of 1,217 *N-*glycosylation sites from 696 *N-*glycoproteins and the detection of 887 desialo-*O-*glycopeptides from 160 *O-*glycoproteins (Figure 2B and 2C and Supplementary Tables 7–8).

In total, 1,310 *N-*glycosylation sites were identified by combining the de-*N*-glycosylated peptides enriched by HILIC SPE and TiO_2_ SPE and pre-fractionated by HILIC HPLC (Supplementary Tables 2, 7). We then compared our coverage of the urinary *N*-glycosylation sites in PCa and BPH urine to a SWATH based identification of the *N*-glycosylation sites reported in normal prostate, non-aggressive, aggressive and metastatic PCa tumor tissues [21]; a substantial overlap of 337 unique glycosylation sites (25%) and 321 *N-*gycoproteins (44%) was observed (Supplementary Figure 2A–2B). This result indicates that almost half of the glycoproteins expressed in prostate tissues appear to be mirrored in the excreted urine. We then compared our urinary *N-*glycoproteome coverage to the *N-*glycoproteome of two PCa cell lines LNCap and PC3 reported by Shah *et al.* [20]. In total, 134 *N-*glycoproteins (11%) were found to be common between our two data sets (Supplementary Figure 2B). Interestingly, the urinary glycoproteins identified in our study had a closer resemblance to the glycoproteome of PCa tissues than the glycoproteome of the PCa cell lines.

### Quantitative comparison of PCa-associated glycan-compositions and lectin blotting analysis

We compared the relative abundance of all intact *N-*glycopeptides grouped by glycan class and terminal features from PCa and BPH urine. In doing this, no significant differences were observed between PCa and BPH with respect to the overall relative abundance of high mannose, afucosylated/asialylated complex/hybrid (C/H), fucosylated complex/hybrid (C/H+Fuc), sialylated complex/hybrid (C/H+NeuAc), and fucosylated/sialylated complex/hybrid (C/H+Fuc/Sia). However, increased intensity of Fuc-containing intact desialo-*O-*glycopeptides was observed (*T*-test, *p* < 0.05) (Supplementary Figure 3).

**Figure 3:**
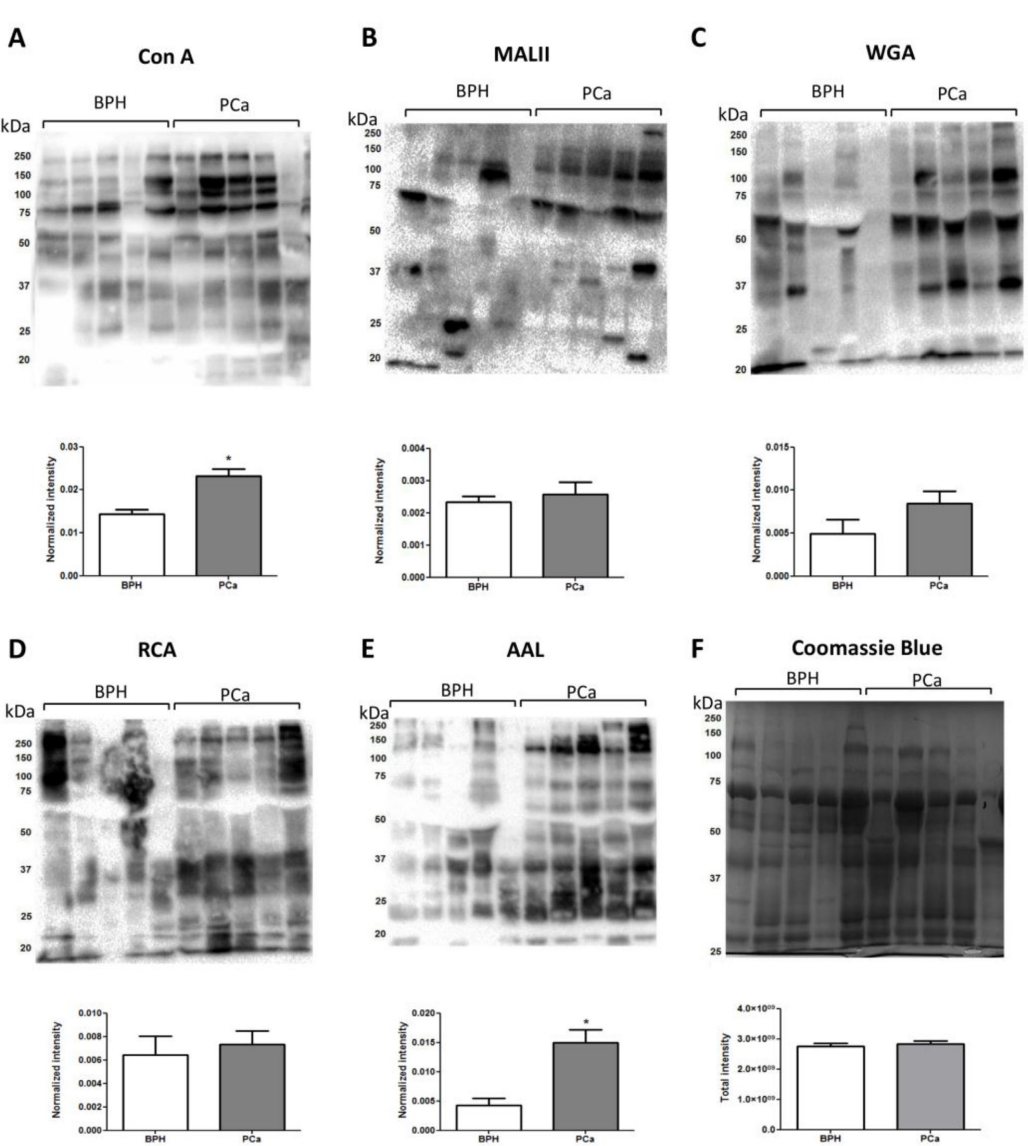
Lectin blotting analysis of PCa and BPH urine Proteins from PCa and BPH urine (20 ug) were separated on SDS-PAGE gels, transferred to PVDF membranes and blotted against Con A (**A**), MALII (**B**), WGA (**C**), RCA (**D**), AAL (**E**) lectins. The intensity of each lane after lectin blotting was normalized by the intensity of the respective sample stained by Coomassie (**F**).

Comparative lectin blotting was also performed using *concanavalin A* (Con A), *Maackia amurensis* lectin (MAL), *wheat germ agglutinin* (WGA), *Aleuria aurantia* lectin (AAL) and *Ricinus communis* agglutinin (RCA) to assess the level of a range of glycoepitopes in PCa and BPH urine in an orthogonal manner. After protein normalization, significantly increased levels of AAL reactivity were observed for the PCa urinary glycoproteome indicating a higher degree of core fucosylation (Figure 3). Slightly higher Con A reactivity was also observed in PCa relative to BPH urine, a feature not reflected in the quantitative LC-MS/MS data of high mannose glycopeptides. However, Con A may also cross-react with paucimannosidic and biantennary complex glycoproteins possibly explaining this discrepancy [35]. Based on these observations, we therefore sought to determine whether site-specific glycosylation instead would display a higher potential of discriminating between urine from PCa and BPH patients.

### Comparative quantitative glycoproteomics to identify unique site-specific glycosylation signatures in PCa urine

The datasets of de-*N-*glycosylated peptides and desialo-*O-*glycopeptides generated after TiO_2_ SPE-based glycopeptide enrichment and HILIC HPLC pre-fractionation as well as the intact *N-*glycopeptides identified after HILIC SPE and TIO_2_ SPE-based glycopeptide enrichments was used to quantitatively compare the glycosylation profile of PCa and BPH urine using TMT reporter ion intensities. In addition, to assess for altered protein levels, the intensities from the non-modified peptides (TiO_2_ SPE flow-through fraction) were also compared between PCa and BPH. The ion intensities of all glycopeptides or non-modified peptides were summed, log2 transformed, and median normalization was applied. One BPH sample showed different outlier behavior when comparing all samples using PCA and was therefore removed. To reveal statistical differences in the abundance of glycopeptides from PCa (*n =* 5) and BPH groups (*n =* 4), the Limma R package was used [36]. The total number of differentially abundant intact *N*-glycopeptides and desialo-*O-*glycopeptides, de-*N*-glycosylated peptides and non-modified peptides, are shown in Figure 2D and Supplementary Table 9.

We then performed unsupervised PCA to assess if these glycopeptides and non-modified peptides were able to discriminate PCa from BPH. Excitingly, all differentially abundant peptides, including the intact *N-* and *O-*glycopeptides, de-*N-*glycosylated peptides and non-modified peptides, were able to almost completely separate the PCa and BPH groups (Supplementary Figure 4A). Using only the non-modified peptides that were significantly changing in abundance, an overlap between PCa and BPH groups was observed. Interestingly, only the differentially abundant intact *N-*glycopeptides and desialo-*O-*glycopeptides separated completely the PCa and BPH groups in the PCA. As a complementary data representation style, we performed clustering analysis using Euclidean distance and a heat map to visualize the altered levels of intact *N-*glycopeptides and desialo-*O-*glycopeptides. Two large clusters of glycopeptides were observed containing 120 over-represented glycopeptides and 106 under-represented glycopeptides in PCa compared to BPH (Supplementary Figure 4B). These clusters perfectly separated the PCa samples from the BPH samples. These results showed that the urinary glycoproteome provided better discrimination between the PCa and BPH groups than the non-modified peptides.

Parallel reaction monitoring (PRM), a targeted proteomics strategy [37], was performed to: 1) confirm the levels of some intact glycopeptides in PCa urine; 2) increase the number of peptide-spectrum matches (PSM) for the individual intact glycopeptides to improve the identification confidence and 3) improve the quantification accuracy and ion statistics of intact glycopeptides with aberrant levels in the PCa urinary glycoproteome.

The intact *N-*glycopeptide SVVAPATDG GLNLTSTFLR displaying the triantennary and core-fucosylated sialoglycoform HexNAc(5)Hex(6)Fuc(1)NeuAc(3) from prostaglandin-H2 D-isomerase (PTGDS) was present in 29 PRM-MS/MS spectra within the retention time 31.2–32.9 min. The intact *O-*glycopeptide DLCNFNEQLENGGTSLSEK carrying the glycoform HexNAc(3)Hex(1)Fuc(1) from CD59 glycoprotein (CD59) was present in 24 PRM-MS/MS spectra within the retention time 22.8–24.9 min. In comparison, each of these two glycopeptides were only identified from two acquired MS/MS spectra in a data-dependent acquisition (DDA) mode (Supplementary Tables 4 and 6). The TMT reporter ion intensities from the identified spectra of each glycopeptide were summed and normalized to the sum of all TMT reporter ion intensities from each TMT10plex channel during the entire LC-MS/MS run. Statistical differences were assessed by conventional *t*-test analyses. The increased levels of the HexNAc(5)Hex(6)Fuc(1)NeuAc(3) *N-*glycopeptide from PTGDS and decreased levels of the HexNAc(3)Hex(1)Fuc(1) *O-*glycopeptide from CD59 in PCa compared to BPH urine was confirmed (Supplementary Figure 5). Example of annotated PRM-MS/MS spectra of these two intact *N*- and *O*-glycopeptides are shown in Supplementary Figure 6. Importantly, seven non-modified peptides from CD59 and eight non-modified peptides from PTGDS were identified. Most of these non-modified peptides were unaltered in abundance when comparing between the PCa and BPH urine (Supplementary Table 10). This indicates that the expression levels of these two glycoproteins are likely not altered in PCa, but that the glycosylation of them instead is the molecular feature undergoing regulation.

### Intact *N-*glycopeptide marker panel for PCa diagnosis

To provide a panel of confidently characterized PCa-associated intact glycopeptides, we selected only intact *N-*glycopeptides with significant changes in abundance, whose glycosylation sites were also identified in the de-*N-*glycosylated peptide fractions and whose glycan compositions were commonly known human serum glycoforms reported in UniCarbKB [38]. Besides, we considered only intact glycopeptides displaying fold changes greater than 1.3 or less than 0.77 when comparing PCa to BPH urine. A total of 56 intact *N-*glycopeptides matched these criteria (Table 1). Information of the relative levels of their corresponding glycoproteins was obtained using quantitative LC-MS/MS data of the non-modified peptide fraction (Supplementary Table 10).

**Table 1:** Differentially abundant intact *N*-glycopeptides identified in PCa and BPH urine (PCa = 5, BPH = 4, limma test, *q* < 0.25)

Protein ID			Intact N-glycopeptides							de-N-gycosylated glycosylated peptides		Non-modified peptides	
UniProtKB ID	Gene	Protein	N-glycan composition				*q*-value	Ratio* PCa/BPH	Direction	N-Glycosylation site (de)	Ratio* PCa/BPH	# peptides	Av. ratio* PCa/BPH
			HexNAc	Hex	Fuc	NeuAc							
P01042	*KNG1*	Kininogen-1	6	7	1	4	0.22	0.47	Down PCa	LNAENN(de)ATFYFK	1.10	20	1.7
P01857	*IGHG1*	Immunoglobulin heavy constant gamma 1	4	5	1	1	0.20	2.18	Up PCa	EEQYN(de)STYR	1.15	10	0.8
P01859	*IGHG2*	Immunoglobulin heavy constant gamma 2	4	4	1		0.20	0.49	Down PCa	EEQFN(de)STFR	0.74	3	0.5
P01877	*IGHA2*	Immunoglobulin heavy constant alpha 2	5	3	1		0.20	1.58	Up PCa	TPLTAN(de)ITK	0.68	2	1.3
P02760	*AMBP*	Protein AMBP	4	5		1	0.23	1.60	Up PCa	YFYN(de)GTSMACETF QYGGCMGNGNNFVTEK	1.90	24	2.4
			5	6			0.23	2.47	Up PCa	YFYN(de)GTSMACETF QYGGCMGNGNNFVTEK	1.90		
			6	6	1		0.20	3.32	Up PCa	YFYN(de)GTSMACETF QYGGCMGNGNNFVTEK	1.90		
P02765	*AHSG*	Alpha-2-HS-glycoprotein	4	5		2	0.20	0.33	Down PCa	AALAAFNAQNN(de) GSNFQLEEISR	1.38	8	1.7
			4	6	1		0.20	2.56	Up PCa	AALAAFNAQNN(de) GSNFQLEEISR	1.38		
P02788	*LTF*	Lactotransferrin	4	7		1	0.23	0.51	Down PCa	TAGWNIPMGLLFN (de)QTGSCK	0.52	23	0.7
P05090	*APOD*	Apolipoprotein D	7	6			0.24	1.40	Up PCa	ADGTVNQIEGEATPVN (de)LTEPAK	2.78	6	0.6
P05155	*SERPING1*	Plasma protease C1 inhibitor	4	5		2	0.20	2.76	Up PCa	ASSNPN(de) ATSSSSQDPESLQDR	0.92	5	1.5
P07911	*UMOD*	Uromodulin	5	7		3	0.20	0.35	Down PCa	FALLMTNCYATPSSN (de)ATDPLK	0.87	29	1.4
			4	6		1	0.20	0.14	Down PCa	FALLMTNCYATPSSN (de)ATDPLK	0.87		
			5	4		1	0.20	0.30	Down PCa	FALLMTNCYATPSSN (de)ATDPLK	0.87		
			6	7		3	0.23	0.46	Down PCa	FALLMTNCYATPSSN (de)ATDPLK	0.87		
			6	7		2	0.25	0.54	Down PCa	FALLMTNCYATPSSN (de)ATDPLK	0.87		
			6	5	1	3	0.20	0.58	Down PCa	FALLMTNCYATPSSN (de)ATDPLK	0.87		
			9	4	1		0.20	0.76	Down PCa	FALLMTNCYATPSSN de)ATDPLK	0.87		
			7	6			0.20	1.55	Up PCa	FALLMTNCYATPSSN (de)ATDPLK	0.87		
			5	6		2	0.23	0.59	Down PCa	CNTAAPMWLN (de)GTHPSSDEGIVSR	1.03		
			5	6	1	3	0.20	0.19	Down PCa	QDFN(de) ITDISLLEHR	1.16		
			6	7		4	0.20	0.48	Down PCa	QDFN(de) ITDISLLEHR	1.16		
P08185	*SERPINA6*	Corticosteroid-binding globulin	4	5		2	0.20	0.32	Down PCa	AQLLQGLGFN(de) LTER	0.67		
P10909	*CLU*	Clusterin	4	5	2	1	0.23	1.61	Up PCa	LAN(de) LTQGEDQYYLR	0.80	6	1.6
			5	6		3	0.20	3.01	Up PCa	KEDALN(de)ETR	1.46		
			4	5		2	0.23	2.46	Up PCa	EDALN(de)ETR	1.56		
	*CD59*	CD59 glycoprotein	2	2			0.23	1.48	Up PCa	TAVN(de)CSSDFDACLITK	1.18	7	2.1
			5	5	1	1	0.22	1.54	Up PCa	TAVN(de)CSSDFDACLITK	1.18		
			4	5		2	0.21	1.67	Up PCa	TAVN(de)CSSDFDACLITK	1.18		
			5	5	1		0.22	1.70	Up PCa	TAVN(de)CSSDFDACLITK	1.18		
P13987			4	6	1	1	0.23	1.71	Up PCa	TAVN(de)CSSDFDACLITK	1.18		
			5	5	1	1	0.25	1.73	Up PCa	TAVN(de)CSSDFDACLITK	1.18		
			5	5	1		0.24	1.73	Up PCa	TAVN(de)CSSDFDACLITK	1.18		
			4	6	1	1	0.24	1.78	Up PCa	TAVN(de)CSSDFDACLITK	1.18		
			4	6	2		0.24	1.91	Up PCa	TAVN(de)CSSDFDACLITK	1.18		
P28300	*LOX*	Protein-lysine 6-oxidase	4	5	1	2	0.20	2.43	Up PCa	DPGAAVPGAAN(de)ASAQQPR	1.21	1	1.9
			4	6	2		0.20	2.82	Up PCa	DPGAAVPGAAN(de)ASAQQPR	1.21		
			4	5	1	1	0.23	1.84	Up PCa	RDPGAAVPGAAN(de)ASAQQPR	1.54		
P29622	*SERPINA4*	Kallistatin	4	5		1	0.21	0.34	Down PCa	FLN(de)DTMAVYEAK	0.85		
			4	5	2		0.21	0.34	Down PCa	FLN(de)DTMAVYEAK	0.85		
P41222	*PTGDS*	Prostaglandin-H2 D-isomerase	5	7		2	0.25	0.45	Down PCa	WFSAGLASN(de)SSWLR	0.34	8	2
			4	5	1	2	0.20	0.32	Down PCa	WFSAGLASN(de)SSWLR	0.34		
			5	6	2		0.20	0.63	Down PCa	WFSAGLASN(de)SSWLR	0.34		
			5	6	1	3	0.21	0.47	Down PCa	SVVAPATDGGLN(de)LTSTFLR	0.93		
			6	7	1	2	0.20	0.48	Down PCa	SVVAPATDGGLN(de)LTSTFLR	0.93		
			5	7	1	1	0.22	0.64	Down PCa	SVVAPATDGGLN(de)LTSTFLR	0.93		
P41271	*NBL1*	Neuroblastoma suppressor of tumorigenicity 1	4	5	1	2	0.22	1.76	Up PCa	N(de)ITQIVGHSGCEAK	1.24	1	3.6
Q07954	*LRP1*	Prolow-density lipoprotein receptor-related protein 1	2	2	1		0.24	1.97	Up PCa	QSGDVTCN(de)CTDGR	1.65	2	1.9
Q14508	*WFDC2*	WAP four-disulfide core domain protein 2	5	7		3	0.20	1.95	Up PCa	TGVCPELQADQN(de)CTQECVSDSECADNLK	1.30	6	1.6
			4	6		1	0.22	1.90	Up PCa	TGVCPELQADQN(de)CTQECVSDSECADNLK	1.30		
			6	3	1	2	0.20	2.12	Up PCa	TGVCPELQADQN(de)CTQECVSDSECADNLK	1.30		
Q6GTX8	*LAIR1*	Leukocyte-associated immunoglobulin-like receptor 1	5	6	1	1	0.24	2.10	Up PCa	STYN(de)DTEDVSQASPSESEAR	0.96	2	1.8
			4	5	1	1	0.21	2.16	Up PCa	STYN(de)DTEDVSQASPSESEAR	0.96		
Q96FE7	*PIK3IP1*	Phosphoinositide-3-kinase-interacting protein 1	4	6		2	0.23	0.65	Down PCa	CLNWLDAQSGLASAPVS GAGN(de)HSYCR	0.57	6	1.7
			5	6	1	3	0.25	0.63	Down PCa	CLNWLDAQSGLASAPV SGAGN(de)HSYCR	0.57		

Only intact *N*-glycopeptides detected with significant differences in abundance (ratio PCa/BPH >1.3 and <0.76), carrying a glycan composition reported in UniCarbKB and with the glycosylation site confirmed in the de-*N*-glycosylated peptide fraction are displayed in this table.

^*^Ratio was calculated by the average of normalized TMT reporter intensities from the PCa (*n =* 5) and BPH (*n =* 4) samples.

Table 1 shows site-specific glycoforms that are more/less abundant in PCa independently of the protein level. For example, protein AMBP (AMBP) was clearly more abundant at the protein level (as detected by ten non-modified peptides with increased levels, Supplementary Table 9) and site-specific glycan regulation was observed by the increased relative abundance of three intact glycopeptides covering two *N*-glycosylation sites in PCa urine (Table 1). On the other hand, apolipoprotein D (APOD) was less abundant in PCa (two non-modified peptides with decreased levels, Supplementary Table 9), but increased levels of site-specific glycosylation (i.e. HexNAc(7)Hex(6)) were observed. In contrast, LTF was found to be both less abundant at the protein level (six under-represented non-modified peptides in PCa) and with respect to the *N-*glycosylation site occupancy as evaluated by the under-representation of the de-*N*-glycosylated peptide TAGWNIPMGLLFDQTGSCK and the site-specific hybrid glycoform HexNAc(4)Hex(7)NeuAc(1) in PCa. Most of the glycoproteins showed no changes at the protein level, but were aberrantly changing with respect to their site-specific glycosylation levels e.g. CLU, LOX, SERPINA4, WFDC2, LAIR1, PIK3IP1 (Table 1). This indicates that the glycosylation machinery, and not the protein translation and secretion, of the cells producing these cancer-associated glycoforms is significantly impacted by the malignant processes associated with PCa.

Some glycopeptides of the urinary glycoproteome were putatively identified with terminal NeuGc. Although being a non-human type of sialic acid, NeuGc may, however, be a component of human glycoproteins arising from exogenous building blocks in particular in glycoproteins of cancer cell origin [39]. These putative NeuGc-containing glycopeptides were manually checked for the presence of the corresponding oxonium ions i.e. *m/z* 308/290 in the corresponding HCD-MS/MS spectra. All of the glycopeptides showed diagnostic ions for NeuAc (*m/z* 274/290) instead of NeuGc. The molecular mass of the compositions HexNAc(5)Hex(6)Fuc(1)NeuAc(2)NeuGc(1), HexNAc(5)Hex(6)Fuc(1)NeuAc(1)NeuGc(1), HexNAc(5)Hex(6)Fuc(1)NeuAc(2)NeuGc(1), HexNAc(4)Hex(5)Fuc(1)NeuAc(1)NeuGc(1) are identical to the mass of HexNAc(5)Hex(7)NeuAc(3), HexNAc(5)Hex(7)NeuAc(2), HexNAc(5)Hex(7)NeuAc(3), HexNAc(4)Hex(6)NeuAc(2), respectively. As verified by manual annotation, the NeuAc-containing glycoforms were found to be the correct glycan composition in all of these cases, Table 1. Besides, the composition HexNAc(4)Hex(5)NeuGc(1) was found to be incorrectly identified due to an concomitant oxidation of the peptide. In fact, the correct composition was HexNAc(4)Hex(5)NeuAc(1).

**Figure 4:**
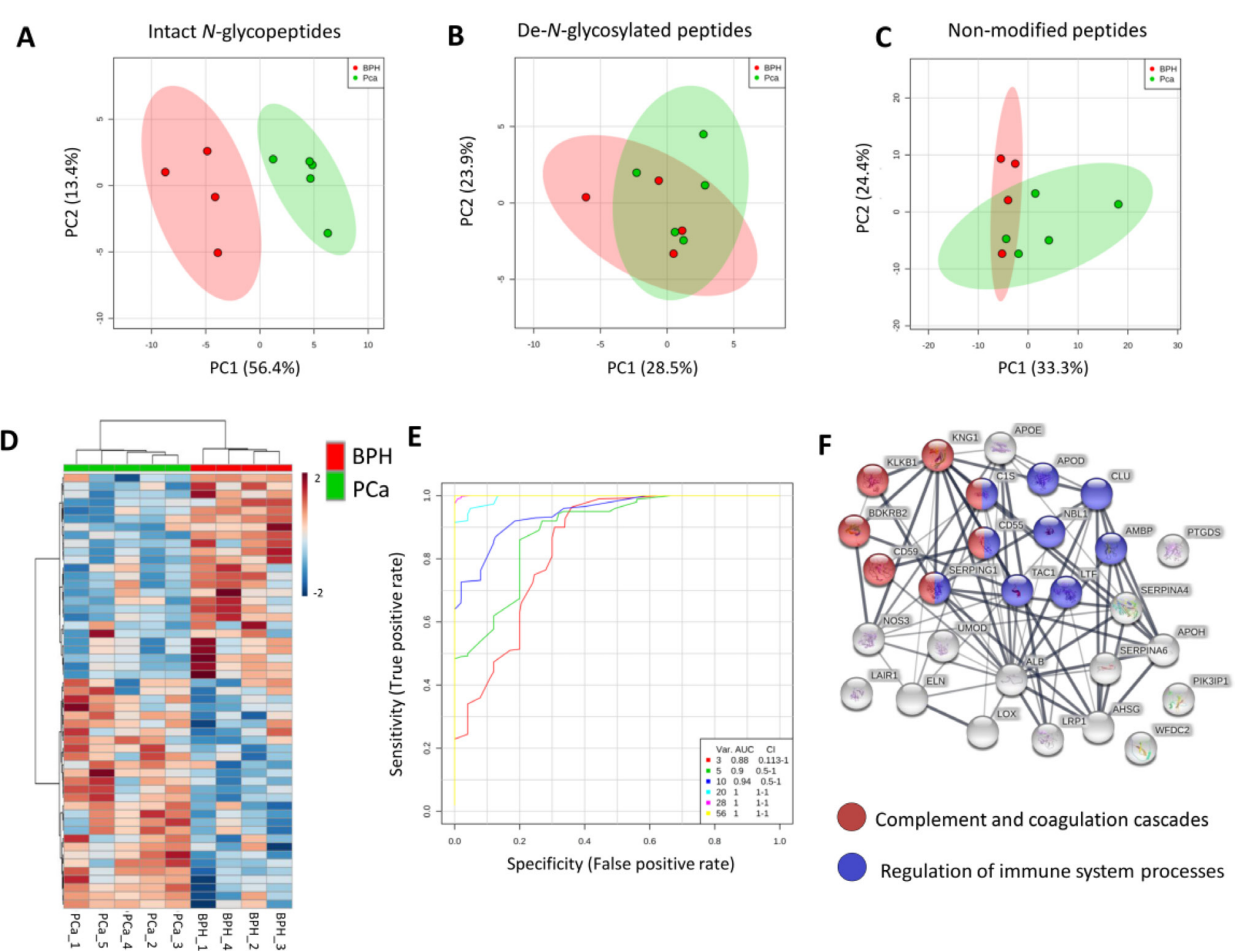
Intact *N-*glycopeptides as candidate biomarkers for PCa detection (**A**) Principal component analysis (PCA) using a panel of 56 *N-*glycopeptides with aberrant levels in PCa relative to BHP (Table 1). (**B**) PCA using a set of 27 deamidated peptides covering the same glycosylation sites as identified with intact *N-*glycopeptide analysis (Table 1). (**C**) PCA using a set of non-modified peptides from the glycoproteins that carried the panel of differentially abundant intact *N-*glycopeptides (Table 1). (**D**) Clustering of intact *N-*glycopeptides displaying altered expression in PCa is shown as a heat map after applying Euclidean distance. (**E**) ROC curve analysis was performed based on partial least squares discriminant analysis (PLS-DA) using the panel of PCa-associated intact *N-*glycopeptides (Table 1).(**F**) Network analysis using the list of glycoproteins which carried the differentially abundant intact *N-*glycopeptides (Table 1). Significant enriched KEGG pathway complement and coagulation cascades (FDR < 0.05) and regulation of immune system response (Gene Ontology, FDR < 0.05) are colored in blue and red, respectively.

PCA was performed using a panel of 56 intact *N-*glycopeptides identified with high confidence. Complete segregation was observed between PCa and BPH (Figure 4A). Interestingly, both the abundance of the glycosylation sites (as measured by the de-*N-*glycosylated peptides) and proteins (measured by the non-modified peptides) from the glycoproteins that were present at different levels in PCa vs BHP were not able to discriminate between the two diseases (Figure 4B–4C). These results indicate that most PCa-specific glyco-features are not due to quantitative changes in the protein or site occupancy level, but arise from altered abundance of specific glycoforms of the individual urinary glycoproteins from the PCa patients.

The PCa-specific panel of 56 intact *N-*glycopeptide was also visualized by a heat map. Unsupervised clustering was able to accurately separate the PCa and BPH donors groups (Figure 4D).

The *N*-glycopeptide panel was also evaluated by ROC curve analysis. The AUC was calculated using combinations of 3, 5, 10, 20, 28 and 56 selected *N*-glycopeptides within the entire glycopeptide panel (56 *N*-glycopeptides). Using 20, 28 or 56 intact *N-*glycopeptides, an AUC of 1 was obtained demonstrating high specificity and sensitivity of these urinary glycopeptides in the discrimination between the PCa patients from the BHP patients (Figure 4E).

The relationship between the glycoproteins that carried aberrant *N*-glycosylation in PCa relative to BHP was explored in a protein-protein network analysis to search for over-represented biological functions and pathways. Significant enrichment of pathways pertaining to the complement and coagulation cascade (KEGG pathway) and a regulation of immune system response (Gene Ontology Biological process) were observed in the network of glycoproteins carrying aberrant glycosylation in PCa (Figure 4F).

In support of our finding, many of the glycoproteins in our panel were already shown in previous studies to be altered in PCa or involved in tumor growth and development. For example: AMBP for PCa diagnosis [40], CD59 associated with PCa progression [41–43], CLU as a therapeutic target against PCa [44] and predictor for PCA recurrence [45], and PTGDS was found in increased concentration in urine from PCa patients [40]. The association of these glycoproteins to PCa in these previous studies was established based on the protein expression level. In contrast, we show here for the first time, that site-specific glycan compositions are also altered in this set of glycoproteins in PCa.

## DISCUSSION

In this study, 1,310 de-*N-*glycosylated peptides, 954 intact *N-*glycopeptides and 887 desialylated but otherwise intact *O-*glycopeptides belonging to a total of 788 glycoproteins were identified. This deep coverage should be evaluated in the light of past efforts in this space including a study by Halim *et al.* [46], which showed the characterization of 58 *N-* and 63 *O-*glycopeptides from 53 urinary glycoproteins, Saraswat *et al.* [47] who reported 51 *N-*glycosylation sites belonging to 37 glycoproteins from the urinary exosomes and our own past study of urine i.e. Kawahara *et al.* [27] reporting 472 unique *N-*glycosylation sites covering 256 urinary glycoproteins. Thus, this present study clearly represents the deepest coverage of the *N-* and *O-*glycoproteome of human urine to date.

One of the technical issues associated with urine analysis is the high inter- and intra-individual variability [6], which was previously shown to account for 48% and 66% of CV variation, respectively [48]. In addition, the multi-step sample preparation and LC-MS/MS acquisition are likely sources of technical variation [49]. We took advantage of the available TMT labeling technology, which not only allowed multiplexing of samples to reduce the LC-MS/MS acquisition time, but also minimized the technical variation from the sample handling [49], especially the peptide enrichment and pre-fractionation, while simultaneously facilitating more accurate quantification of glycopeptides and non-modified peptides [50]. Due to the relative low number of biological replicates and high variance between the individuals, our study was limited to a non-stringent threshold of *q* < 0.25 for the peptide FDRs to determine differentially abundant glycoproteins in PCa and BPH urine. However, by validating the levels of specific intact glycopeptides by PRM-MS/MS we significantly enhanced the confidence in these observations.

An important limitation of the chosen workflow is that, in complex mixtures, co-isolation of multiple precursor ions is the most acknowledged limitation of quantitative proteomics using isobaric labels. This shortcoming reduces the precision and accuracy of the quantification [51, 52]. It was demonstrated that due to this interference problem, the actual abundance ratios are typically underestimated [49]. Using precursor intensity fraction (PIF) tool available in MaxQuant [53], we filtered peptides that clearly suffer from co-isolation of other peptide precursor ions.

Glycans facilitate and contribute to many different aspects of tumor progression, including proliferation, invasion, angiogenesis and metastasis [54]. In cancer, protein glycosylation is dynamically regulated due to altered expression of glycan-modifying enzymes [55, 56]. Wang *et al.* [57] showed high expression of α1,6-fucosyltransferase (FUT8) in tumor tissue from patients with metastatic and aggressive primary PCa and was positively correlated with PCa with high Gleason scores. The *N-*glycosylation of proteins was demonstrated previously to be altered in urine [58], tissues [21] and serum [59] from PCa patients as well as in PCa cell lines [20]. Interestingly, using meta-analysis of publicly available transcriptomic data from different PCa studies, Barfeld *et al.* [60] reported a concise 33 gene signature with biological enrichment for protein glycosylation, which discriminates between PCa and BPH across multiple transcript detection platforms and sample types. Meany *et al.* [59] reported that direct analysis of PSA *N*-glycosylation in sera may be able to improve the sub-optimal specificity of PSA as a PCa marker. Altered glycosylation of PSA isolated from PCa serum and/or seminal plasma relative to PSA glycosylation from controls was repeatedly observed by Tabares *et al.* [61], Llop *et al.* [62] and Ohyama *et al.* [63]. Collectively, these studies clearly support the association of altered of protein glycosylation with PCa. Although glycoproteomics studies have previously been used to explore PCa [20–22], the quantitative information relating to site-specific *N-* and *O-*glycosylation and the glycan compositions were not reported in those studies.

We demonstrated that differentially abundant glycopeptides discriminate more accurately between PCa and BPH groups than differentially abundant non-modified peptides. Thus, we argue that a selected panel of glycopeptides, but not their corresponding non-glycosylated peptides from the same protein, may serve as candidate biomarkers for PCa detection. We demonstrated that the malignant processes associated with PCa are more frequently reflected directly in the glycosylation of proteins as oppose to the aberrations in the expression level or the glycosylation site occupancy of those proteins. Although we were limited to investigating a relative small patient cohort, the potential of intact glycopeptides to stratify patient groups can be explored in follow-up studies using larger cohort of patients once the glycoproteomics technology matures further allowing more streamlined data collection and interpretation of a larger patient cohort.

Among the PCa-associated glycopeptides, we targeted the *N-*glycosylation of prostaglandin-H2 D-isomerase (PTGDS) and the *O-*glycosylation of CD59 using PRM analysis. Interestingly, Davalieva *et al.* [40] and Ahmad *et al.* [7] showed that these two glycoproteins are over-expressed in the urine of PCa patients. Herein, we complement their observations by reporting that PTGDS and CD59 carry aberrant glycosylation at defined positions in PCa urine.

CD59 is a glycosyl-phosphatidylinositol (GPI)-anchored cell membrane glycoprotein that inhibits complement-mediated cell lysis by preventing full assembly of the membrane attack complex (MAC) on host cells, and thus might confer immune resistance to tumor cells [42]. High expression of CD59 protein was associated with higher Gleason scores and higher pT stages in PCa [41]. It was demonstrated that prostasomes, which are secretory granules produced, stored, and released by the glandular epithelial cells of the prostate, had higher expression of CD59 than those of normal cells [64]. CD59 glycosylation was previously characterized and several roles for the glycans, including spacing and orienting CD59 on the cell surface and protecting the molecule from proteases were proposed based on structural data [65].

Prostaglandin-H2 D-isomerase was identified in human seminal plasma [66]. Prostaglandin D2 (PGD2) and prostaglandin D2 synthase (PTGDS) is involved in the regulation of testis tissue differentiation [67, 68]. PGD2, which is synthesized by PTGDS in many organs, has been implicated as a signaling molecule in the mediation or regulation of various biological processes, including tumorigenic process in PCa [69]. It was shown that PTGDS activity is defined by post-translational modifications, and point mutation in a glycosylation site (Asn51) inhibited L-PGDS-induced apoptosis and caspase 3 activity [70]. Interestingly, we identified a significant lower abundance in the Asn51 glycoform HexNAc(4)Hex(5)Fuc(1)NeuAc(2) and HexNAc(5)Hex(6)Fuc(2) in PCa compared to BPH urine (Table 1).

The quantitative analysis of intact glycoproteins opens novel avenues for assessing the dysregulation of glycoproteins in PCa and tie such information with aberrant glycoprotein function as drivers or passive by-products of tumour development and progression.

The quantification of specific peptides glycoforms is challenging due to the low ionization efficiency of glycan-modified peptides, as well as the lack of glycopeptides reference spectra. Moreover, multiple glycoforms (micro-heterogeneity) are detected as separate glycopeptides, thus diminishing the signal intensity for each glycopeptide form. Selected reaction monitoring (SRM) and parallel reaction monitoring (PRM) are currently the leading methods for targeted MS-based quantification of proteins. Recently, SRM was applied to quantify candidate non-modified peptides biomarkers in expressed prostatic secretions (EPSs) from PCa and control patients [71]. Song *et al.* [72] applied MRM to quantify intact glycopeptides in depleted human blood serum using the glycans oxonium ions as transitions. However, to this date quantification of intact glycopeptides using targeted MS approaches is still poorly explored. We showed PRM is a useful approach to quantify intact glycopeptides. The number of MS/MS spectra recorder increased beyond a 20 fold by using PRM for targeted glycopeptide analysis, improving the accuracy of the identification and quantitation. Integrating this approach with synthetic glycopeptides for normalization and absolute quantification would enhance value and utility of the PRM-MS/MS workflow even further.

We found the complement and coagulation cascades to be over-represented pathways in the set of urinary glycoproteins carrying differentially abundant site-specific glycoforms. Interestingly, the same pathway was observed to be enriched in the meta-analysis from Barfeld *et al.* [60] which contained differentially expressed genes between localized PCa and benign prostate tissue, thus demonstrating that biological processes occurring in prostatic tissues may be reflected in urine.

Different coagulation disorders were reported during prostatic carcinoma evolution [73]. The most frequent coagulation complication is the disseminated intravascular coagulation (DIC), which is a result of the release of pro-coagulant substances, such as tissue factors, into the bloodstream [74]. This coagulation disorder was reported in many patients with PCa [75–77]. We identified seven glycoproteins displaying aberrant site-specific glycosylation that are related to complement and coagulation cascade pathway. The set of differentially abundant site-specific glycoforms belonging to these glycoproteins can be explored as candidate biomarkers for coagulation disorders that may be present during PCa progression.

Additionally, recent reports suggest that the complement elements can promote tumor growth in the context of chronic inflammation, immunosuppression, angiogenesis, and cancer cell signaling [78–80]. Manning *et al.* also showed that human PSA, via its chymotrypsin-like serine protease activity, can modulate the complement system through degradation of iC3b to produce new C3 degradation fragments and through degradation of the complement protein C5, thereby inactivating the complement cascade [81].

In summary, this study reports several innovative approaches advancing our pursuit of reliable and sensitive urinary biomarker for PCa discovery and stratification from BPH by 1) providing the largest coverage of the urinary *N*- and *O*-glycoproteome to date, 2) providing a panel of 56 *N-*glycopeptides derived from aberrantly expressed glycoproteins in PCa urine representing an exciting collection of potential candidate biomarkers for discrimination of PCa from BHP and by 3) achieving confident structural characterization including the peptide carrier identity, site annotation, and glycan composition as well as quantitative validation of the panel of glycopeptide candidate markers by the application of innovative LC-MS/MS and PRM approaches of intact glycopetides. This study opens new promising avenues for using urinary glycoproteins, a hitherto largely untapped resource, as candidate biomarkers for PCa detection.

## MATERIALS AND METHODS

### Urine collection, protein extraction and peptide generation

The study was approved by the ethics review board of the Instituto do Câncer do Estado de São Paulo (ICESP), under the protocol n°784/15. Written informed consents were obtained from all participants. The methods were performed in accordance with the approved guidelines and regulations.

The first voided urine after prostate biopsy was collected prospectively from men who were admitted for prostate biopsies based on serum PSA levels ≥ 4.0 ng/ml and/or abnormal DRE. All men had received study information, and they had signed the informed consent. In this study, a total of 12 subjects were included, of whom 6 were validated to have PCa based on biopsy examination by a trained pathologist. The men were asked to void, and the first ∼30 ml of urine was collected and immediately cooled on ice. The samples were centrifuged at 3,000 × g for 10 min at 4° C. The cleared supernatant (∼2–20 ml) was further filtered using 0.22 μm filters (Millipore, Billerica, MA). Two milliliters of filtered urine was then concentrated using 10 kDa cutoff filters (Millipore, Billerica, MA) and stored immediately at −20° C until further use. The total protein amount was determined using Qbit fluorometric quantitation (Thermo).

### Protein digestion and desalting

Proteins (100 µg) were reduced by addition of dithiothreitol (DTT) to a final concentration of 10 mM and incubated for 30 min at 30° C. Proteins were alkylated prior to digestion by the addition of iodoacetamide (IAA) to a final concentration of 40 mM and incubated for 30 min in the dark at room temperature. To quench the reaction, DTT was added to a final concentration of 10 mM. Porcine trypsin (1:50, w/w, sequence-grade, Promega) was added, and the mixture incubated overnight at 37° C. The reaction was stopped with the addition of 1% TFA and the resulting peptides were purified using a primed Oligo R3 reversed phase SPE micro-column and dried [29].

### Isobaric peptide labeling with TMT

Peptides originating from ∼100 µg protein from each urine sample were dissolved in 100 µl 100 mM TEAB and labeled individually with TMT-10plex mass tags (Thermo) according to the manufacturer’s instructions. Briefly, each TMT10plex tag (0.8 mg) was resuspended with 41 μl neat anhydrous acetonitrile and added to each of the peptide samples and the mixture incubated for 1 h at room temperature after vigorous vortexing for 5 min. To quench the reaction, 8 μl 5% (v/v) hydroxylamine was added per sample followed by 15 min incubation at room temperature. After TMT labelling, all peptide samples were mixed 1:1 (w:w), divided into two aliquots (∼500 µg each) and purified on a primed Oligo R3 reversed phase SPE micro-column.

### Glycopeptide enrichment using titanium dioxide (TiO_2_) SPE

One of the aliquots from the TMT-labeled peptide samples (∼500 µg) was used for sialoglycopeptide enrichment using TiO_2_ SPE according to Larsen *et al.* [28] and Palmisano *et al.* [29]. Samples were resuspended in 1 ml loading buffer containing 1 M glycolic acid, 80% acetonitrile (ACN), 5% TFA. The samples were incubated with TiO_2_ beads (GL Sciences, Japan, 5 µm) using a total of 0.6 mg TiO_2_ beads per 100 µg peptides and shaken at room temperature for 15 min. The suspension was centrifuged at 1,000 × g for 1 min and the supernatant transferred to another vial with a fresh batch of TiO_2_ beads (containing half the amount of TiO_2_ beads as initially used) and shaken at room temperature for 15 min. The two batches of TiO_2_ beads were washed with 100 µl loading buffer and centrifuged at 1,000 × g for 1 min. The supernatant was discarded and the beads washed with 100 µl washing buffer 1 containing 80% ACN in 1% aqueous TFA (both v/v) and centrifuged at 1,000 × g for 1 min. The supernatant was discarded and the beads were washed with 100 µl washing buffer 2 containing 20% ACN in 0.2% aqueous TFA (both v/v) and centrifuged at 1,000 × g for 1 min. The supernatant was again discarded and the beads were dried in a vacuum centrifuge for 5 min. The bound peptides were eluted by resuspending the TiO_2_ beads in 100 µl elution buffer containing 1% (v/v) aqueous ammonium hydroxide and by vortexing the beads for 15 min. The mixtures were then centrifuged at 1,000 × g for 1 min, the peptide-containing supernatant transferred to separate tubes and the eluted peptides were aliquoted and dried by vacuum centrifugation. This produced an enriched sialoglycopeptide fraction.

### Glycopeptide enrichment using hydrophilic interaction liquid chromatography (HILIC) SPE

The second aliquot of TMT-labeled urinary peptides (∼500 µg) was reconstituted in 100 µl loading and washing buffer containing 80% ACN in 1% aqueous (both v/v) TFA. Peptides were loaded onto a primed custom-made HILIC SPE micro-column according to Mysling *et al.* [30] packed with PolyHYDROXYETHYL A™ resin (PolyLC Inc) onto a supporting C8 disk (Empore) in a p200 pipette tip. The flow-through fraction was collected. The HILIC SPE columns were then washed in 100 µl loading and washing buffer and the wash fraction was collected and combined with the first flow-through fraction for separate downstream analysis. The enriched glycopeptides were eluted with 50 μl 0.1% (v/v) TFA followed by 50 μl 25 mM NH_4_HCO_3_ and finally 50 μl 50% (v/v) ACN; these three elute fractions were combined. The flow through and the combined elute fraction were dried by vacuum centrifugation and purified on a primed Oligo R3 reversed phase SPE micro-column, aliquoted and dried again by vacuum centrifugation. This produced an enriched glycopeptide fraction.

### PNGase F and sialidase treatment

An aliquot of the TiO_2_ or HILIC SPE enriched (sialo)glycopeptides was resuspended in 50 mM TEAB, pH 8.0 and simultaneously de-*N*-glycosylated using 500 U *N*-glycosidase F (PNGase F) (New England Biolabs, Ipswich, MA) and de-*O*-sialylated using 0.1 U broad-specificity sialidase A (Prozyme Hayward, CA). The PNGase F and sialidase treatment were carried out concomitantly for 12 h at 37° C with the purpose of achieving efficient de-*N*-glycosylation while rendering the *O*-glycopeptide more amendable to LC-MS/MS characterization. After incubation, the de-*N*-glycosylated and de-*O*-sialylated peptides were purified on a primed Oligo R3 reversed phase SPE micro-column, dried by vacuum centrifugation and reconstituted in 50 µl 0.1% formic acid prior to LC-MS/MS analysis.

### HILIC HPLC pre-fractionation

The desialylated glycopeptides enriched by TiO_2_ as well as the flow through containing asialoglycopeptides were resuspended in 90% ACN in 0.1% (both v/v) aqueous TFA and injected onto an HPLC column (320 μm ID × 170 mm length) packed in-house using TSKgel Amide-80 HILIC 5 μm resin (Tosoh Bioscience) connected to an Agilent 1200 micro-HPLC instrument. Briefly, samples were suspended in solvent B containing 90% ACN in 0.1% (both v/v) aqueous TFA. Peptides were loaded onto a 320 μm peak HILIC LC column and eluted at 6 μl/min by decreasing the solvent B from 100–60% over 42 min [82]. Fractions were automatically collected in a 96 well plate at 1 min intervals after UV detection at 210 nm and dried by vacuum centrifugation, combined in 8–16 relevant fractions according to the corresponding UV profile and stored at −20° C until LC–MS/MS analysis.

### Mass spectrometry analysis

The pre-fractionated peptides were resuspended in 0.1% FA and automatically loaded on a trap column (2 cm × 100 µm inner diameter) custom packed with ReproSil-Pur C18 AQ 5 µm resin (Dr. Maisch, Ammerbuch-Entringen, Germany). A total of ∼1 µg of peptide was injected. The peptides were separated at 250 nl/min on an analytical column (17 cm × 75 µm) packed with 3 µm resin of the same kind operated in reversed phase chromatography mode using an EASY-nanoLC system (Thermo Fisher Scientific, Odense, Denmark). The mobile phases were 95% ACN in 0.1% (both v/v) aqueous FA (solvent B) and aqueous 0.1% (v/v) FA (solvent A). The linear gradient of solvent A increased from 3% to 28% over 52 min, 28–47% over 5 min, 45–100% over 5 min and 8 min at 100% B. The nanoLC was connected to a Q Exactive HF Hybrid Quadrupole-Orbitrap mass spectrometer (Thermo Fisher Scientific) operating in positive ion mode and using data dependent acquisition. The Orbitrap acquired the full MS scan with an automatic gain control (AGC) target value of 3 × 10^6^ ions and a maximum fill time of 100 ms. Each MS scan was acquired at high-resolution 120,000 full width half maximum (FWHM) at *m/z* 200 in the Orbitrap with a *m/z* range of 400–1600 Da. The 10 most abundant precursor ions were selected from each MS full scan for higher-energy collision-induced dissociation (HCD) fragmentation using normalized collision energy (NCE) of 29% if they were at least doubly charged. Fragmentation was performed at high resolution (60,000 FWHM) for a target of 1 × 10^5^ product ions and a maximum injection time of 200 ms using a precursor isolation window of *m/z* 1.2 and a dynamic exclusion of 30 s after a single isolation and fragmentation of a given precursor.

For parallel reaction monitoring (PRM), full MS scans were acquired at 60,000, AGC target was 1 × 10^6^, maximum injection time was 100 ms and the *m/z* range was 700–1800. HCD fragmentation was performed using an inclusion list with the following parameters: 60,000 resolution for an ion target of 1 × 10^5^ and a maximum injection time of 150 ms using an isolation window of *m/z* 1.6 and a fixed lower *m/z* of 110.

### Analysis of intact *N-*glycopeptides

Intact glycopeptide HCD-MS/MS fragment spectra from each sample were searched against defined protein and glycan search spaces using the Byonic software v2.6.46 (Protein Metrics, https://www.proteinmetrics.com/) [83]. Searches were performed with the following fixed modifications: precursor ion mass tolerance of 10 ppm, product ion mass tolerance of 0.02 Da, carbamidomethylation of Cys, and strict trypsin specific cleavage with a maximum of two missed cleavages per peptide. Searches were also conducted with the following variable modifications: oxidation of Met (+15.994 Da), TMT (+229.163) at N-term and K, and *N-*glycosylation of sequon-localized Asn with the predefined *N*-glycan database of 309 mammalian *N*-glycans without sodium or *O*-glycosylation of Thr and Ser with the predefined *O*-glycan database containing 78 common mammalian *O*-glycans without sodium available within Byonic. To narrow the protein search space only the list of glycoproteins identified by the detection of sequon-containing de-*N-*glycosylated peptides generated by PNGase F treatment were used in the protein database for the identification of intact *N*-glycopeptides. For intact *O*-glycopeptides, a protein database was composed by glycoproteins annotated in UniProtKB. All searches were filtered to <1% false discovery rate (FDR) at the protein level and 0% at the peptide level by using a decoy database of the reversed sequences of the list of proteins used for the original (forward) protein search space [84]. We conducted strict filtering criteria, where only glycopeptides identified with high score ID was considered (PEP 2D scores < 0.001). PEP 2D scores less than 0.001 means that there is less than 0.1% chance of having an incorrectly annotated peptide (but not necessarily correct annotation of the site and glycan composition) of the glycopeptides [27]. Further manual validation was performed for glycopeptides with “difficult-to-assign” glyco-features e.g. NeuGc-containing glycopeptides were validated by searching for the diagnostic ions pertaining to NeuGc *m/z* 308.090/290.090 at a mass tolerance of 10 ppm [85]. Using oxonium ions to validate glycopeptides should be performed carefully since co-isolation of different intact glycopeptides within the same *m/z* isolation window occurs especially in complex peptide samples. This was observed for some intact *N-*glycopeptides, which were tentatively assigned as non-NeuAc containing glycopeptides, but with a clear presence of *m/z* 274/292 oxonium ions in the corresponding HCD-MS/MS spectra, useful diagnostic ions for NeuAc-containing glycopeptides (Supplementary Figure 7).

### Protein identification and glycosylation site analysis

For protein identification and glycosylation site analysis, LC-MS/MS raw files were imported into MaxQuant version 1.5.2.8 [53]. The database search engine Andromeda [86] was used to search the HCD- and CID-MS/MS spectra against a database composed of the reviewed UniProtKB human protein database (release April 15, 2015; 45,185 entries) with a precursor ion tolerance level of 4.5 ppm and product ion mass accuracy thresholds of 20 ppm and 0.5 Da for HCD-MS/MS and CID-MS/MS, respectively. For the reporter fragment ions, the 10-plex TMT was included in the search parameters and enzyme specificity was set to trypsin with a maximum of two missed cleavages. Carbamidomethylation of Cys (+57.021 Da) was set as a fixed modification, and oxidation of Met (+15.994 Da), deamidation of Asn and Gln (+0.984 Da) and protein *N-*terminal acetylation (+42.010 Da) were selected as variable modifications. Five variable modifications per peptide were allowed. All identifications were filtered in order to achieve 1% PSM and Protein FDR. The reporter ion intensities for each PSM with PIF (precursor ion fraction) > 0.75 were calculated using MaxQuant. The quantitation of the identified proteins was determined by reporter ion intensities using at least 1 razor/unique peptide.

After excluding peptides identified as potential contaminants or appearing in the reverse database, we manually filtered the peptides containing the deamidation sites located within the conserved *N*-glycosylation motif i.e. NxS/T/C where x ≠ P.

### Bioinformatic analysis

Bioinformatic analyses were performed using Perseus v.1.5.4.1 which is available within MaxQuant. The reporter ion intensities from redundant peptides were summed. The quantitation of the identified de-*N*-glycopeptides and non-modified peptides was performed by normalizing the reporter ion intensities by log2 conversion and subtracting them by the log2 mean of each sample. The first biological replicate of the BPH sample did not cluster together with the rest of the samples as assessed by principal component analysis (PCA) and was therefore removed. Statistical tests were carried out using the Limma R package [36], which have been shown to perform well for proteomics data in the case of low replicate numbers and missing values [87]. Resulting *p*-values were corrected for multiple statistical testing and converted into false discovery rates of *q* < 0.25.

PCA was constructed in the web-based chemometrics platform MetaboAnalyst version 2.0 [88]. Protein-protein interaction networks and enrichment analyses were performed using the STRING software [89].

### Lectin blotting analysis

An aliquot of 20 μg urinary proteins were separated by discontinuous electrophoresis (stacking gel consisting of 4% polyacrylamide and resolving gel consisting of 12% polyacrylamide) for 1 h. The separated proteins were then transferred to a polyvinylidene fluoride (PVDF) membrane by wet electroblotting using the Tris/glycine transfer buffer overnight at 4°C. PVDF membranes were blocked in TBS-T and 5% BSA at room temperature for 1 h. The membranes were incubated with the following lectins (all from Vector Laboratories, Burlingame, CA, USA) in TBST for 16 h at a dilution of 1:2000 (v:v) *concanavalin A* (Con A), *Maackia amurensis* lectin (MAL), *wheat germ agglutinin* (WGA), *Aleuria aurantia* lectin (AAL) and *Ricinus communis* agglutinin (RCA).

Then membranes were washed three times for 5 min each with TBS-T and incubated with streptavidin-HRP conjugate (Vector Laboratories, Burlingame, CA, USA, 1:5,000 for Con A and 1:2,000 for AAL, MAL, WGA and RCA) at room temperature for 1 h followed by three washes with TBS-T. The PVDF membranes were analyzed with Western Blotting Analysis System Bio-Rad Image Lab™ Software (BI*O-*RAD). The subsequent densitometry analysis of the resulting bands was performed with the Image J Software or Image Lab.

## SUPPLEMENTARY MATERIALS FIGURES AND TABLES























## Abbreviations

PSA: Prostate specific antigen
PCa: prostate cancer
BPH: benign prostatic hyperplasia
HILIC: hydrophilic interaction liquid chromatography
LC-MS/MS: liquid chromatography–mass spectrometry
GS: Gleason score
SPE: solid phase extraction
TiO2: titanium dioxide
PCA: principal component analysis
Con A: concanavalin A
MAL: Maackia amurensis lectin
AAL: Aleuria aurantia lectin
RCA: Ricinus communis agglutinin
PLS-DA: partial least squares discriminant analysis
PRM: Parallel reaction monitoring
PSM: peptide-spectrum matches
