# Supplementary material for: Distinct urinary glycoprotein signatures in prostate cancer patients
Source: Oncotarget. 2018 Sep 4;9(69):33077–97. doi: 10.18632/oncotarget.26005 (PMC6145689; doi:10.18632/oncotarget.26005)
Supplement: Supplementary file 1 [file oncotarget-09-33077-s001.pdf]

Distinct urinary glycoprotein signatures in prostate cancer patients

SUPPLEMENTARY MATERIALS

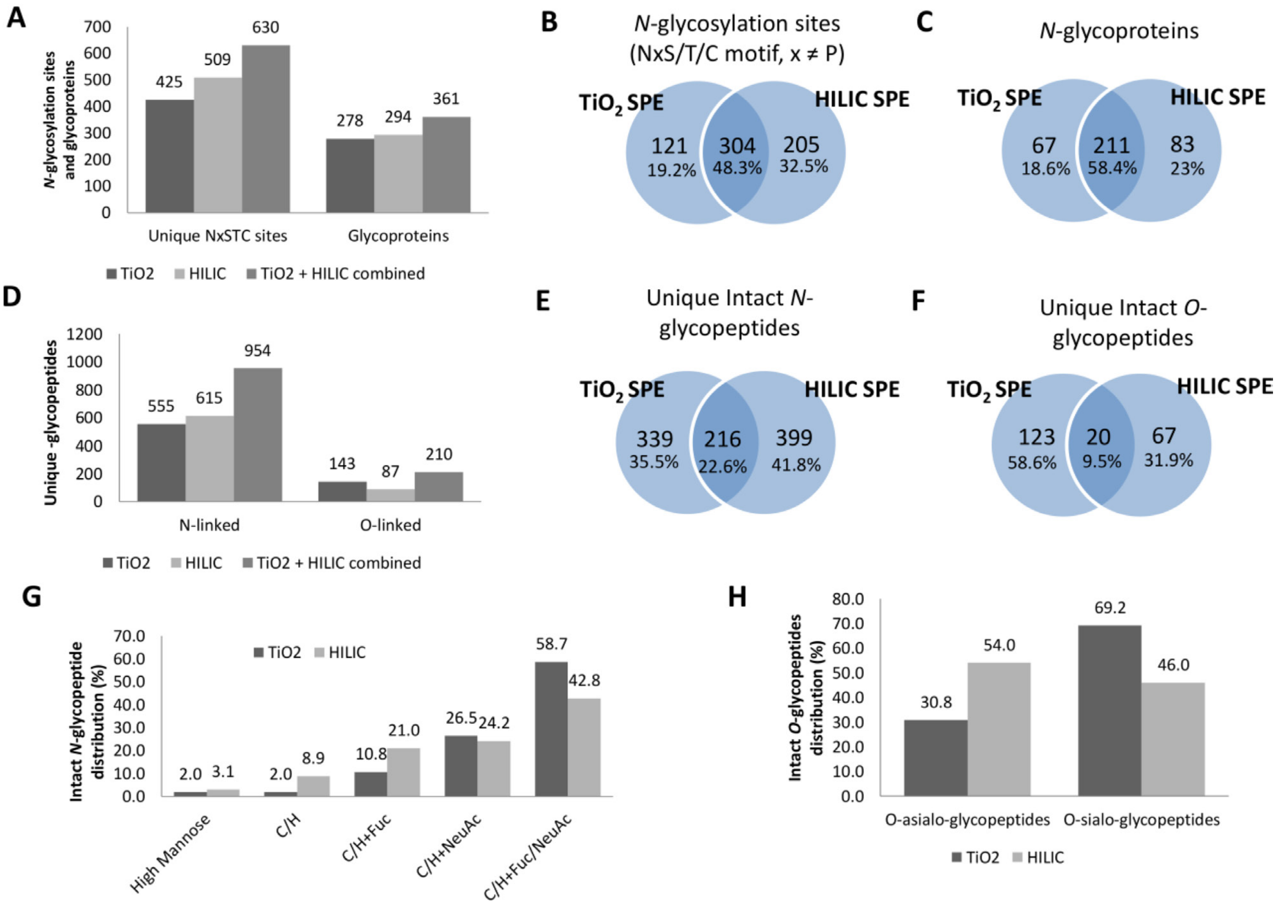

Supplementary Figure 1: TiO_2 and HILIC SPE based glycopeptide detection in human urine (regardless of PCa/BHP status). Distribution and comparison of (**A–C**) *N*-glycosylation sites (de-*N*-glycosylated peptides) and *N*-glycoproteins and (**D–F**) unique intact *N*-glycopeptide and *O*-glycopeptides identified in human urine by LC-MS/MS after glycopeptide enrichment using TiO_2 or HILIC SPE. Only deamidation sites within NxS/T/C motifs (x ≠ P) were considered. (**G**) Distribution of unique intact *N*-glycopeptides identified according to the class (high mannose or complex/hybrid (C/H)) and features (sialylation and fucosylation). (**H**) Distribution of the identified intact *O*-glycopeptides according to presence (sialo) or absence (asialo) of sialic acid residues.

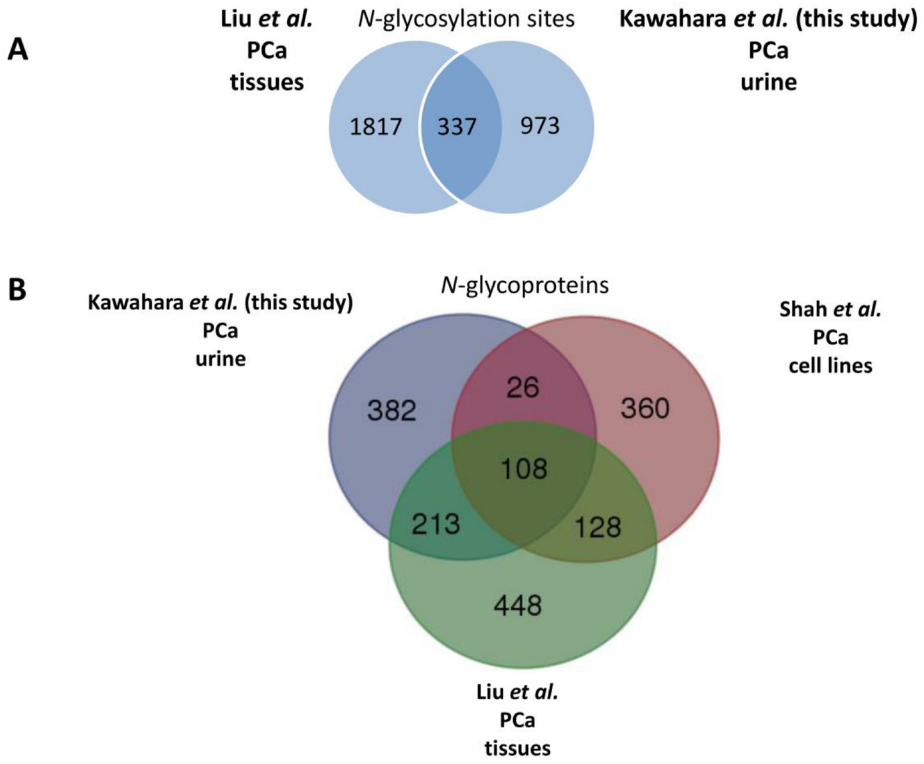

Supplementary Figure 2: Comparison of *N*-glycosylation sites and *N*-glycoproteins identified by LC-MS/MS in PCa urine (this study) and previously reported PCa studies. (**A**) overlap between *N*-glycosylation sites identified in PCa tissues (Liu *et al.*) and PCa urine (this study). (**B**) Comparison of *N*-glycoproteins identified in PCa urine (this study), PCa tissues (Liu *et al*.) and PCa cell lines (Shah *et al.*).

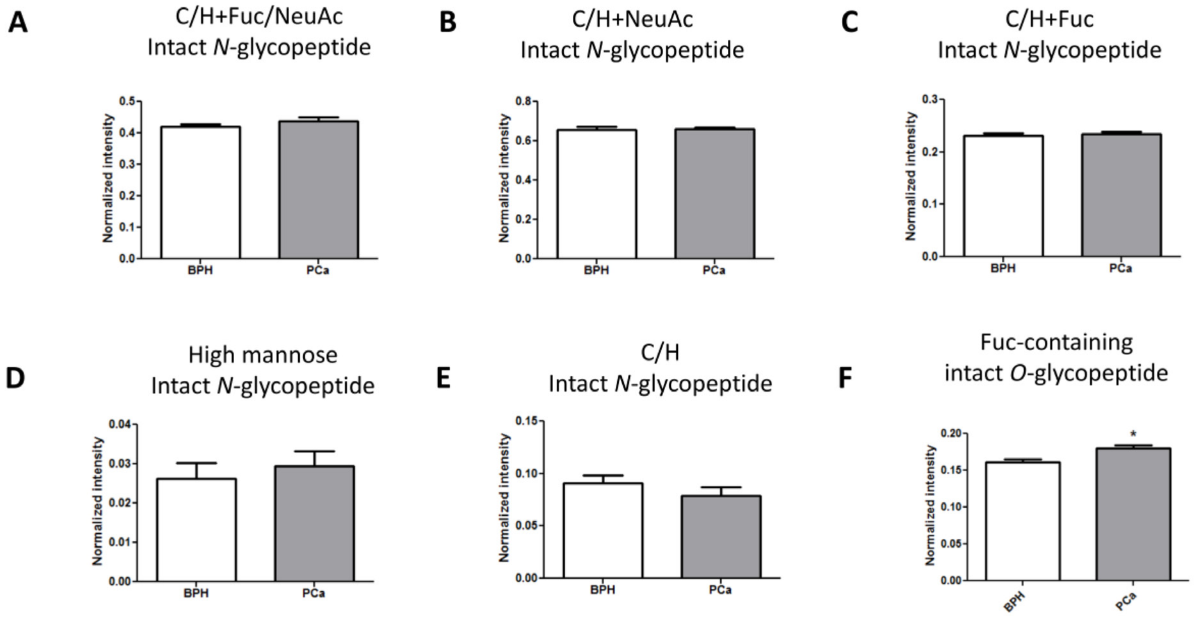

Supplementary Figure 3: Quantitative comparison of glycan compositions in BPH and PCa urine. Intact glycopeptides were grouped by their identified glycan composition class/features and their reported ion intensities were normalized, summed and compared between PCa and BPH. Fucosylated/sialylated complex/hybrid (C/H+Fuc/NeuAc) intact *N*-glycopeptides (**A**), sialylated complex/hybrid (C/H+NeuAc) intact *N*-glycopeptides (**B**) fucosylated complex/hybrid (C/H+Fuc) intact *N*-glycopeptides (**C**), high manose intact *N*-glycopeptides (**D**) afucosylated/asialylated complex/hybrid (C/H) intact *N*-glycopeptides (**E**), Fucose-containing intact desialo-*O*-glycopeptides (**F**).

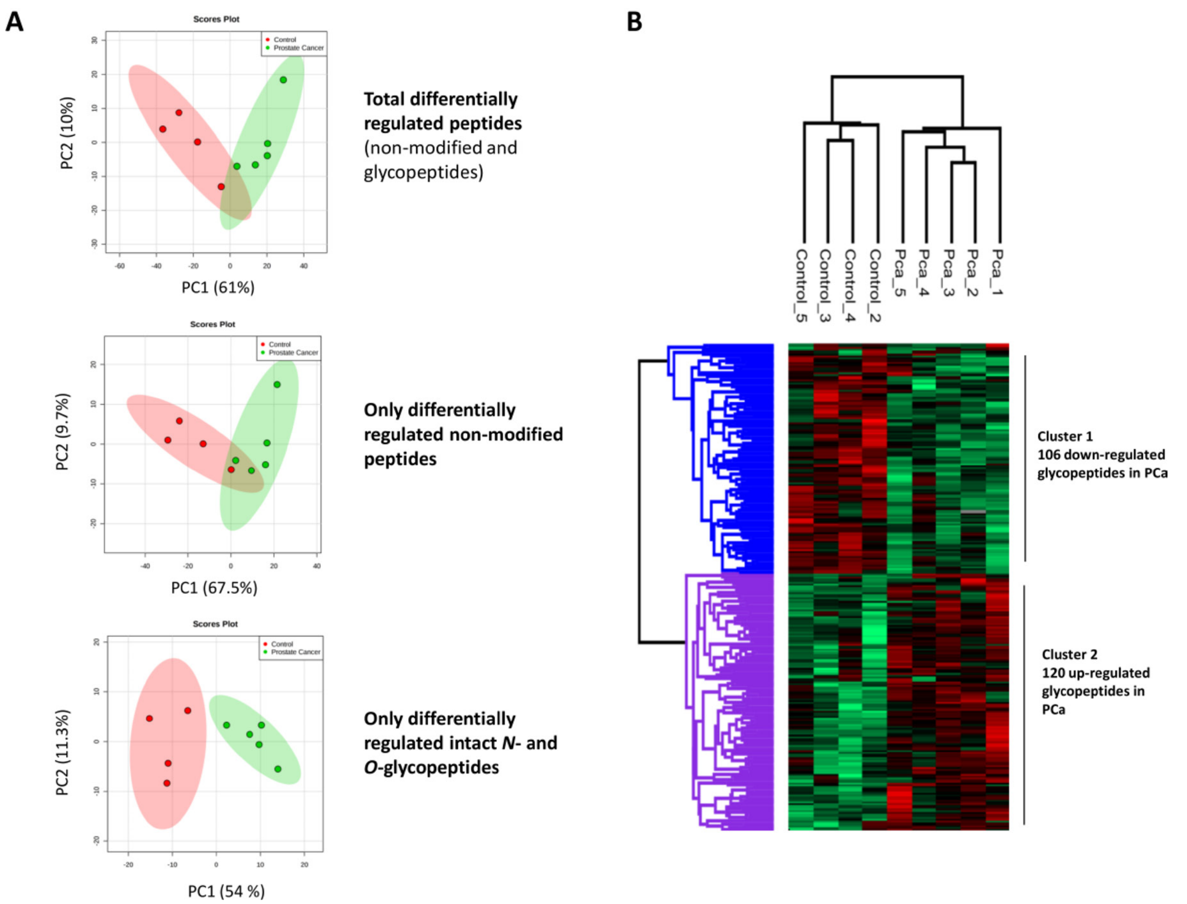

Supplementary Figure 4: Analysis of differentially abundant glycopeptides and non-modified peptides using the normalized TMT report ion intensities from prostate cancer (PCa) and control urines. (**A**) Principal component analysis using the set of differentially regulated intact glycopeptides and/or non-modified peptides. (**B**) Clustering of significant regulated glycopeptides is shown as a heat map after applying Euclidean distance.

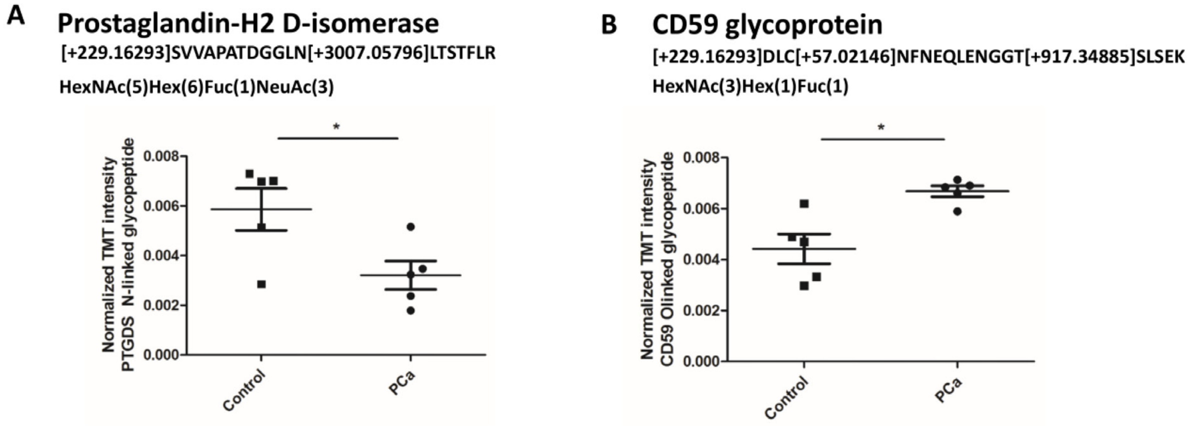

Supplementary Figure 5: PRM analysis of O- and N-linked intact glycopeptide in TMT labeled urine samples. (**A**) PRM analysis of N-linked intact glycopeptide from prostaglandin-H2 D-isomerase (PTGDS), precursor m/z = 1289.5665 and charge +4. (**B**) PRM for O-linked intact glycopeptide from CD59 glycoprotein (CD59), precursor m/z, 826.1100, charge +4. The individual TMT reporter ions from the identified MS/MS spectra were summed and normalized by the sum of all reporter ions intensities quantified for the respective channel. *T*-test was applied and significant difference between prostate cancer and control was considered if $p < 0.05$ (*).

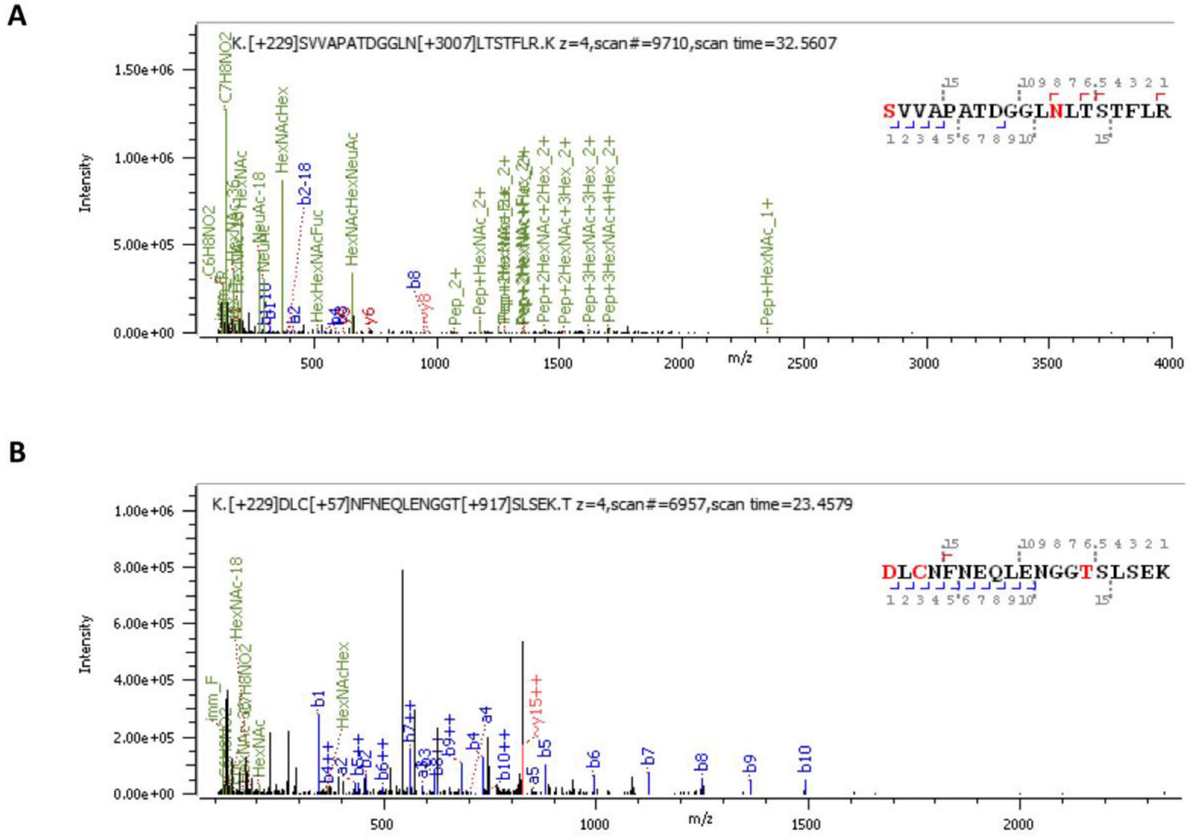

Supplementary Figure 6: (**A**) Example of annotated MSMS spectrum for an N-linked glycopeptides for Prostaglandin-H2 D-isomerase (Uniprot ID: P41222, gene name: PTGDS, m/z = 1289.5665, charge +4, PEP 2D score = 1.2E-6). (**B**) Example of annotated MSMS spectrum for an O-linked glycopeptides for CD59 glycoprotein (Uniprot ID: P13987, gene name: CD59, m/z = 826.1236, charge +4, PEP 2D score = 4.3E-11).

A

R.[+229.16293]YFYN[+2336.85109]GTSMAC[+57.02146]ETFQYGGC[+57.02146]MGNGNNFVTEK[+229.16293].E
HexNAc(6)Hex(6)Fuc(1)
>sp|P02760|AMBP_HUMAN Protein AMBP OS=Homo sapiens GN=AMBP PE=1 SV=1
Retention time: 69.02
Scan: 16515

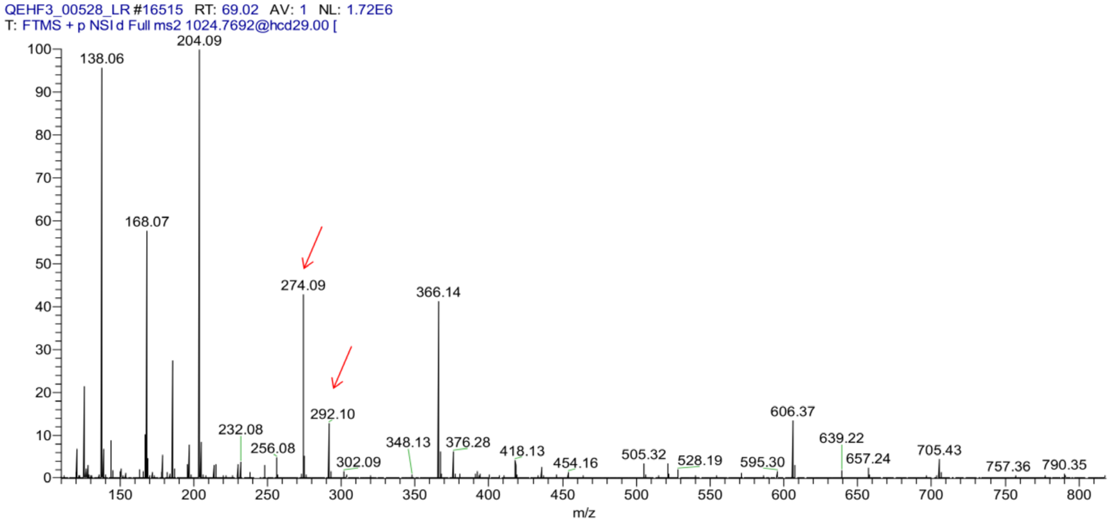

B

K.[+229.16293]TAVN[+1971.71889]C[+57.02146]SSDFDAC[+57.02146]LITK[+229.16293].A
HexNAc(5)Hex(5)Fuc(1)
>sp|P13987|CD59_HUMAN CD59 glycoprotein OS=Homo sapiens GN=CD59 PE=1 SV=1
Retention time: 68.06;68.1
Scan: 16270;16280

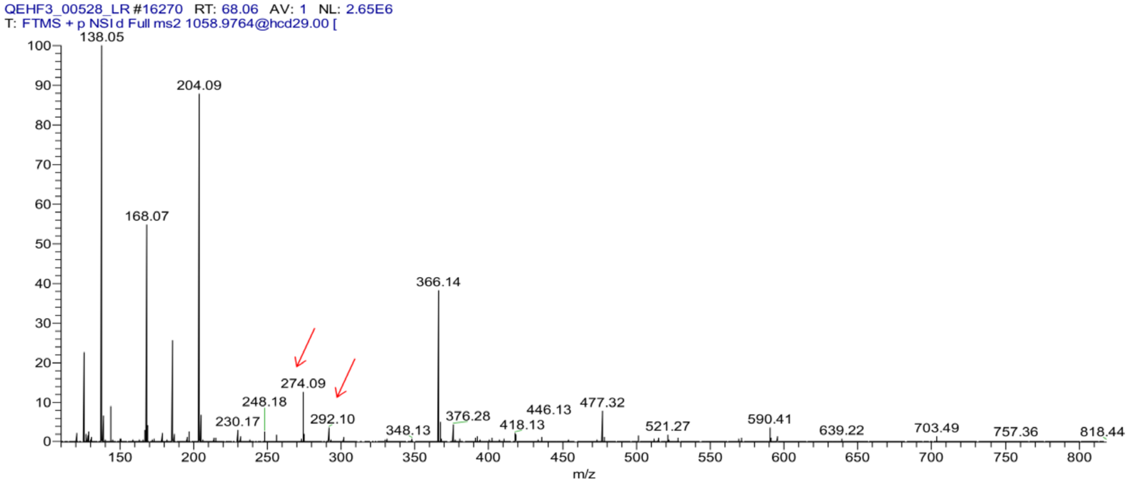

Supplementary Figure 7: Example of intact N-glycopeptide assigned as non-NeuAc containing glycopeptides, but with a clear presence of m/z 274/292 oxonium ions (arrows) in the corresponding HCD-MS/MS spectra. (**A**) MS/MS spectrum of the intact glycopeptide with the peptide sequence: YFYNGTSMACETFQYGGCMGNGNNFVTEK and the glycan composition: HexNAc(6) Hex(6)Fuc(1) belong to the protein AMBP. (**B**) MS/MS spectrum of the intact glycopeptide with the peptide sequence TAVNCSSDFDACLTK and the glycan composition: HexNAc(5)Hex(5)Fuc(1) belonging to the CD59 glycoprotein. The asparagine in bold corresponds to the N-linked glycosylation site.

Supplementary Table 1: Deamidated *N*-glycosylation sites (NxSTC) identified by LC-MS/MS followed by MaxQuant database search using TiO2 SPE enrichment. See Supplementary_Table_1

Supplementary Table 2: Deamidated *N*-glycosylation sites (DxSTC) identified by LC-MS/MS followed by MaxQuant database search using HILIC SPE enrichment. See Supplementary_Table_2

Supplementary Table 3: Intact *N*-glycopeptides identified by LC-MS/MS followed by Byonic database search using TiO2 SPE enrichment. See Supplementary_Table_3

Supplementary Table 4: Intact *N*-glycopeptides identified by LC-MS/MS followed by Byonic database search using HILIC SPE enrichment. See Supplementary_Table_4

Supplementary Table 5: Intact *O*-glycopeptides identified by LC-MS/MS followed by Byonic database search using TiO2 SPE enrichment. See Supplementary_Table_5

Supplementary Table 6: Intact *O*-glycopeptides identified by LC-MS/MS followed by Byonic database search using HILIC SPE enrichment. See Supplementary_Table_6

Supplementary Table 7: Deamidated *N*-glycosylation sites (NxSTC) identified using TiO2 SPE enrichment followed by HILIC HPLC pre-fractionation. See Supplementary_Table_7

Supplementary Table 8: Intact *O*-glycopeptides identified after TiO2 SPE enrichment, PNGase F de-N-deglycosylation and HILIC HPLC pre-fractionation. See Supplementary_Table_8

Supplementary Table 9: Differentially regulated intact *N*- and *O*-glycopeptides, de-*N*-glycosylated peptides and non-modified peptides between PCa and BPH (PCa n = 5, BPH n = 4, limma test, $q < 0.25$). See Supplementary_Table_9

Supplementary Table 10: Non-modified peptides identified in the flow-through fraction after TiO2 enrichment SPE and after HILIC HPLC pre-fractionation. See Supplementary_Table_10
